# Evidence map and gap analysis of metabolic change in pediatric growth hormone deficiency treated with growth hormone

**DOI:** 10.1002/ped4.70050

**Published:** 2026-03-11

**Authors:** Wei Wu, Ningyi Song, Yue Zhao, Caiqi Du, Yuning Zhao, You Wu, Xiaoping Luo

**Affiliations:** ^1^ Department of Pediatrics Tongji Hospital Tongji Medical College Huazhong University of Science and Technology No. 1095, Jiefang Wuhan Hubei 430030 China; ^2^ Medical Affairs Novo Nordisk (Shanghai) Pharma Trading Co., Ltd. No.8, Guangshunnan Beijing 100102 China

**Keywords:** Evidence map, Growth hormone deficiency, Metabolism, Systematic review

## Abstract

**Importance:**

Recombinant human growth hormone (rhGH) improves height in children with growth hormone deficiency (GHD). However, its metabolic effects remain unclear.

**Objective:**

To synthesize evidence regarding the metabolic effects of rhGH or growth hormone (GH) derivatives in GHD, identify knowledge gaps, and highlight future research priorities.

**Methods:**

PubMed, Embase, the Cochrane Library, China National Knowledge Infrastructure, China Biology Medicine disc, and Wanfang databases were searched in July 2023 to identify studies on metabolic effects of GH treatment. Bubble plots were used to visualize GH treatment effects on metabolic parameters according to treatment duration by comparison with baselines, nonpharmacological interventions, and healthy controls. Random‐effects meta‐analyses were conducted for outcomes with inconsistent findings across original studies when randomized controlled trial data were sufficient. The study was registered with INPLASY (INPLASY202450064).

**Results:**

Sixty‐three studies (6158 participants) analyzed the effects of GH treatment on metabolic outcomes in children with GHD. Overall, GH treatment slightly affected glucose levels; lipid effects were inconsistent. GH treatment did not influence bone mineral density, bone mineral content, or parathyroid hormone levels. Most studies showed no significant effects on thyroid hormone levels, body composition, or body mass index (BMI). GH treatment may increase serum asymmetric dimethylarginine and gastrin levels while reducing tumor necrosis factor and serum urea levels.

**Interpretation:**

Glucose and thyroid outcomes are consistent with clinical observations; effects on lipids, calcium, phosphorus, body composition, BMI, and waist‐to‐hip ratio require further validation owing to data inconsistency. New biomarkers are warranted. Further clinical studies are needed in children across age groups, GHD severities, and nutritional statuses.

## Graphical abstract

This evidence map of 63 studies (*n* = 6158) showed growth hormone therapy in children with growth hormone deficiency has neutral glucose effects, mixed lipid outcomes, potential benefits for bone mineral density, bone mineral content, and parathyroid hormone, but generally no impact on thyroid function, body composition, or body mass index.

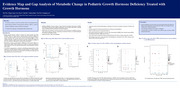

## INTRODUCTION

Human growth hormone (GH) is essential for normal growth and exerts metabolic effects across multiple systems. It acts as an anabolic agent by promoting whole‐body protein synthesis, increasing blood glucose levels, and enhancing lipolysis in adipocytes.^1^ Growth hormone deficiency (GHD), caused by insufficient secretion of human GH by the pituitary gland, is classified as childhood‐ or adult‐onset according to age at presentation. Adults with GHD may develop metabolic abnormalities such as reduced bone density and increased body fat, whereas children primarily experience impaired linear growth. Persistent and untreated GHD can lead to metabolic disorders, cardiovascular disease, and other complications as affected children transition into adulthood, thereby reducing quality of life and life expectancy.^2^ Recombinant human GH (rhGH) or GH derivatives have been shown to improve height outcomes in children with GHD; however, their effects on metabolism, body composition, and related outcomes during transition and adulthood have not been fully characterized.

The 2016 Pediatric Endocrine Society guidelines indicate that the effects of GH therapy on lipid profiles and cardiac function are inconsistent across studies; conclusions regarding bone density and body composition are more consistent, although interstudy variability persists.^3^ These guidelines also emphasize the limited investigation of GH effects on cardiac function, lipid metabolism, body composition, adipokine levels, and peripheral inflammatory markers. According to the 2019 Growth Hormone Research Society guidelines,^4^ the therapeutic goal in children with GHD is to replace deficient GH to support growth and improve metabolic health and well‐being. These guidelines also highlight the importance of evaluating metabolic biomarkers before and after GH treatment to identify novel indicators for assessing diagnosis and therapeutic response. Similarly, the 2019 American Association of Clinical Endocrinologists guidelines state that continued GH therapy during the transition period benefits bone health and body composition.^5^ However, available evidence remains inconsistent; some studies show increased bone density and improved lipid metabolism, whereas others demonstrate no such effects.^6^ Therefore, further investigations are required to clarify the metabolic consequences of GH supplementation in pediatric patients. The above guidelines also emphasize the need for larger, long‐term studies to determine whether metabolic changes persist into adulthood among patients with transitional GHD and whether continued GH replacement improves long‐term overall health.

An evidence map is a systematic methodological approach that visually synthesizes available research evidence in graphical or tabular form. This analytical tool facilitates the identification of existing knowledge gaps and prioritization of future research directions.^7^, ^8^ Through evidence mapping, this review consolidates existing findings to provide empirically grounded guidance for future studies.

## METHODS

### Ethical approval

The analysis was performed in accordance with the Preferred Reporting Items for Systematic Reviews and Meta‐Analysis Extension for Scoping Review (PRISMA‐ScR).^9^ The protocol was registered with INPLASY (INPLASY202450064).

### Eligibility criteria

Eligible studies were randomized controlled trials (RCTs) and observational comparative studies involving patients younger than 18 years who had been diagnosed with GHD and received GH treatment. GHD was defined according to the criteria used in each original study. Treatment involved GH administration without restrictions on dose, frequency, route of administration, device, or duration. Assessed metabolic outcomes included glucose metabolism, lipid metabolism, calcium and phosphorus metabolism, thyroid hormone levels, body composition, body mass index (BMI), waist‐to‐hip ratio (WHR), and novel biomarkers. All studies meeting these criteria were included, regardless of whether metabolic indicators had been reported as primary or secondary outcomes. Metabolic changes were evaluated in three ways: (i) comparison of outcomes before and after GH supplementation; (ii) comparison of outcomes between GH treatment and no treatment; and (iii) comparison of outcomes between children with GHD after GH treatment and healthy children. For comparison (i), all data were derived from RCTs.

Studies published in review, letter, or case report format were excluded, as were those not published in English or Chinese. Studies that did not report the specified metabolic outcomes were also excluded. For comparisons between children with GHD and healthy controls, studies that failed to report baseline or post‐treatment differences between groups were excluded.

### Selection of sources of evidence

We performed a systematic search of electronic databases, including PubMed, Embase, the Cochrane Library, China National Knowledge Infrastructure, China Biology Medicine disc, and Wanfang, on July 25, 2023 (Table S1). We identified additional literature through manual searching. Two reviewers independently screened the studies. Disagreements were resolved by discussion, with arbitration by a third reviewer when necessary. A PRISMA flow diagram was constructed to illustrate the study selection process.

### Data extraction process

Data from each study were extracted by one reviewer and double‐checked by another reviewer. Disagreements were resolved by discussion with assistance from a third party if necessary.

### Study quality assessment

Study quality was appraised using the Cochrane Risk of Bias tool (RoB 1).^10^ All trials included in the meta‐analyses were judged to have low, unclear, or high risk across the standard domains.

### Synthesis of results

Bubble plots were used to present multidimensional information, including measurement time points, metabolic outcome categories, conclusions regarding metabolic changes, and total sample size (bubble size = pooled sample size for studies with a concordant direction of effect at the same time point). Bubble plots were generated with the Sangerbox online mapping tool 3.0. When original studies reported discrepant findings, a meta‐analysis was performed, provided that at least two studies contributed data for the same GH dose stratum and follow‐up interval. RCTs that explicitly reported GH dose and follow‐up duration and provided pre‐ and post‐treatment values for metabolic indices were included. Doses were classified as high, medium, or low, with 0.025–0.035 mg/kg/day (0.07–0.1 IU/kg/day) defined as the medium range; values above or below this range were categorized as high or low, respectively. Subsequent analyses were stratified by follow‐up duration (≤6, 6–12, and ≥12 months). Owing to participant overlap across these intervals, only interval‐specific meta‐analytic estimates were reported; pooled estimates across intervals were not interpreted. All meta‐analyses were conducted with RevMan 5.4 using a random‐effects model. All analyzed outcomes were continuous, and pooled effects were expressed as mean differences with 95% confidence intervals.

## RESULTS

### Results of study selection

**FIGURE 1:**
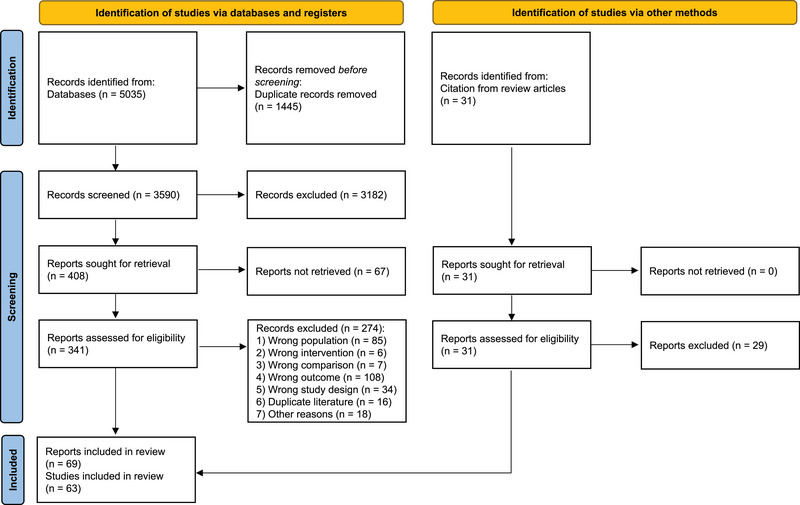
PRISMA flow diagram depicting the full study selection process.

The search identified 5035 relevant citations, of which 63 studies^11^, ^12^, ^13^, ^14^, ^15^, ^16^, ^17^, ^18^, ^19^, ^20^, ^21^, ^22^, ^23^, ^24^, ^25^, ^26^, ^27^, ^28^, ^29^, ^30^, ^31^, ^32^, ^33^, ^34^, ^35^, ^36^, ^37^, ^38^, ^39^, ^40^, ^41^, ^42^, ^43^, ^44^, ^45^, ^46^, ^47^, ^48^, ^49^, ^50^, ^51^, ^52^, ^53^, ^54^, ^55^, ^56^, ^57^, ^58^, ^59^, ^60^, ^61^, ^62^, ^63^, ^64^, ^65^, ^66^, ^67^, ^68^, ^69^, ^70^, ^71^, ^72^, ^73^ involving 6158 participants were included (Figure 1). Some studies contributed to multiple comparisons, yielding 69 comparisons across three categories: 35 assessed metabolic changes before and after GH treatment, 11 compared metabolic outcomes between GH treatment and no medical treatment, and 23 compared metabolic outcomes between children with GHD and healthy controls. Specifically, three studies^28^, ^45^, ^60^ reported data for both before‐versus‐after and GH‐versus‐no‐treatment comparisons, whereas three studies^17^, ^52^, ^62^ reported data for both before‐versus‐after GH treatment and GHD‐versus‐healthy control comparisons.

### Characteristics of included studies

Baseline characteristics of the included studies are presented in Table S2. Studies focusing on specific age groups (e.g., toddlers and preschool children), as well as investigations addressing the causes of GHD, were scarce. Definitions of GHD varied across studies, and nutritional status was infrequently reported. GH dosing regimens and formulations also differed, including daily, weekly, and monthly schedules; most studies adopted daily or weekly administration. Treatment duration ranged from 1 to 60 months, although most studies used treatment periods of ≤12 months.

Most studies did not specify the analytical methods used to quantify metabolic indicators. Assessments of glycolipid metabolism were frequently described as “routine blood tests.” As shown in Table S3, among studies that documented methodological details, the enzyme‐linked immunosorbent assay was the most commonly used technique for metabolic parameter measurement. Evaluations of calcium and phosphate metabolism utilized diverse platforms, including immunoassays, high‐performance liquid chromatography, spectrophotometric colorimetry, and dual‐energy X‐ray absorptiometry.

### Quality assessment results

Owing to limited data availability and substantial clinical heterogeneity across studies, the meta‐analysis was restricted to the synthesis of pre‐ and post‐intervention data from RCTs. Most trials included in the meta‐analyses displayed a low or unclear risk of bias; a small number exhibited a high risk related to allocation concealment and participant blinding (Figures S1 and S2).

### Influence of GH treatment on glucose metabolic outcomes in children with GHD

**FIGURE 2:**
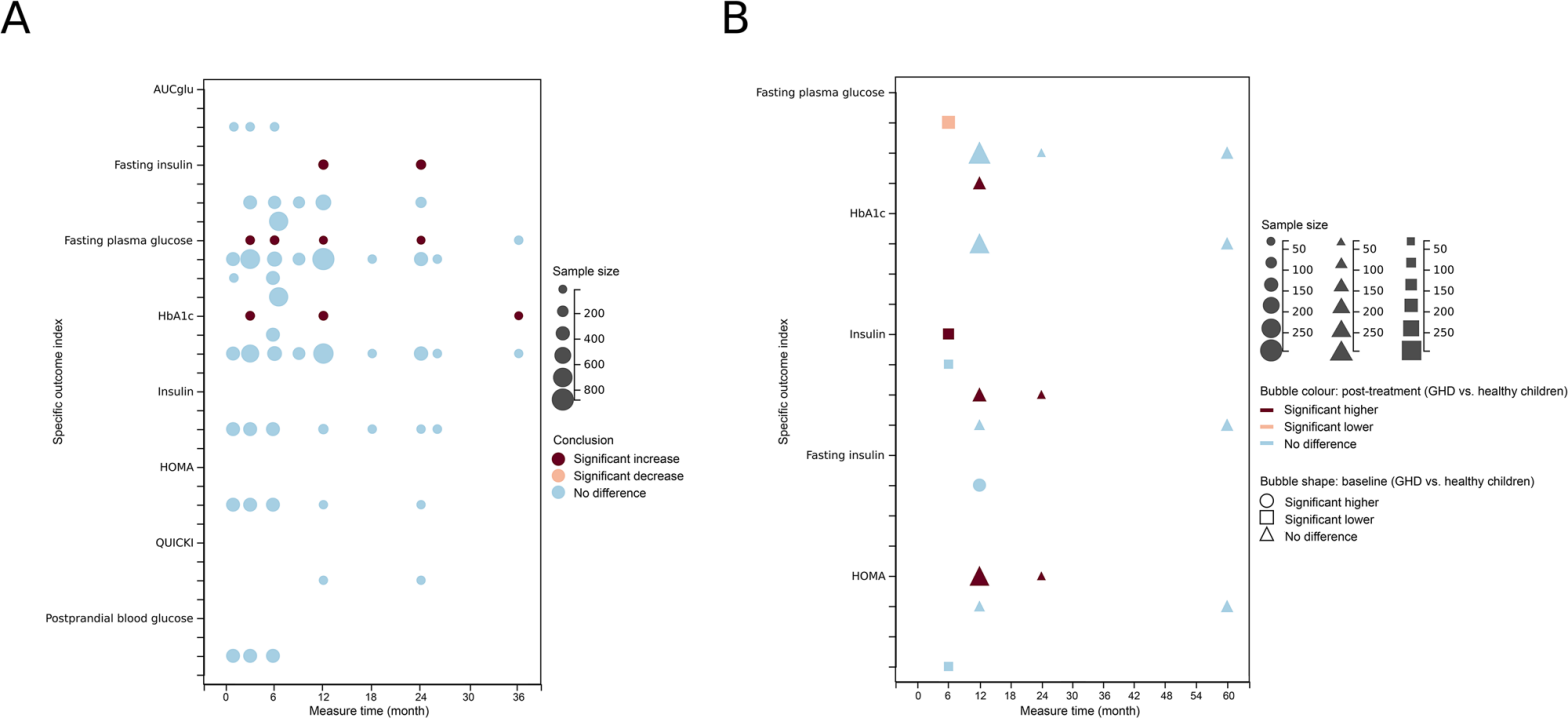
Evidence maps of the effect of GH on glucose metabolic outcomes. (A) Comparison before versus after GH treatment; (B) Comparison between GHD and healthy children. The size of the bubble corresponds to the pooled sample size of studies with concordant effect direction at the given time point. The x‐axis denotes the follow‐up time in months. Abbreviations: AUCglu, area under the glucose curve; GH, growth hormone; GHD, growth hormone deficiency; HbA1c, hemoglobin A1c; HOMA, homeostasis model assessment (insulin resistance); QUICKI, quantitative insulin sensitivity check index (insulin sensitivity).

Nineteen studies^11^, ^12^, ^13^, ^14^, ^15^, ^16^, ^17^, ^18^, ^19^, ^20^, ^21^, ^22^, ^23^, ^24^, ^25^, ^26^, ^27^, ^28^, ^29^ (2364 participants) evaluated glucose levels before and after GH treatment. Overall, GH therapy did not significantly affect the area under the glucose curve (AUCglu), insulin levels, insulin resistance, insulin sensitivity, or postprandial blood glucose levels. However, mixed effects were observed for fasting insulin, fasting glucose, and hemoglobin A1c (HbA1c); some studies demonstrated significant increases, and others showed no significant impact (NSI) (Figure 2A).

Six studies^28^, ^30^, ^31^, ^32^, ^33^, ^34^ (316 participants) compared glucose metabolism in children with GHD between those who received GH treatment and those who did not. The results indicated inconsistent effects of GH therapy on fasting plasma glucose levels, such that some studies displayed significant changes and others showed none. In contrast, GH treatment consistently revealed NSI on HbA1c level, insulin level, insulin resistance, and insulin sensitivity (Table S4).

Eight studies^17^, ^35^, ^36^, ^37^, ^38^, ^39^, ^40^, ^41^ (518 participants) compared glucose metabolism between children with GHD receiving GH treatment and healthy controls. The findings indicated that GH treatment did not significantly affect HbA1c levels, and the difference between children with GHD and healthy controls remained unchanged; however, fasting insulin levels may have been reduced. In contrast, effects on fasting plasma glucose, insulin levels, and insulin resistance were inconsistent; studies showed either an increase or NSI (Figure 2B).

Across the evidence map for the three comparison types, GH treatment consistently showed no significant effects on AUCglu, postprandial glucose levels, or insulin sensitivity. In contrast, other indicators demonstrated either inconsistent or non‐significant findings (Figure 2 and Table S4).

Meta‐analysis of 12 studies^11^, ^14^, ^15^, ^16^, ^17^, ^19^, ^21^, ^22^, ^23^, ^26^, ^29^, ^42^ indicated that GH treatment affected blood glucose levels in a manner consistent with clinical experience and existing evidence. Fasting glucose modestly increased (medium‐dose GH: 0.19–0.40 mmol/L across follow‐up intervals; high‐dose GH: 0.37–0.43 mmol/L); all values remained within the normal range. For HbA1c, medium‐ and high‐dose GH showed a statistically significant difference only at follow‐up durations of ≥12 months, with a modest mean increase of approximately 0.13%. Fasting insulin increased by 1.15 mIU/L with medium‐dose GH during follow‐up of ≤6 months and by 2.64 mIU/L (medium dose) and 4.00 mIU/L (high dose) after follow‐up of ≥12 months (Figures S3–S9).

### Influence of GH treatment on lipid metabolic outcomes in children with GHD

**FIGURE 3:**
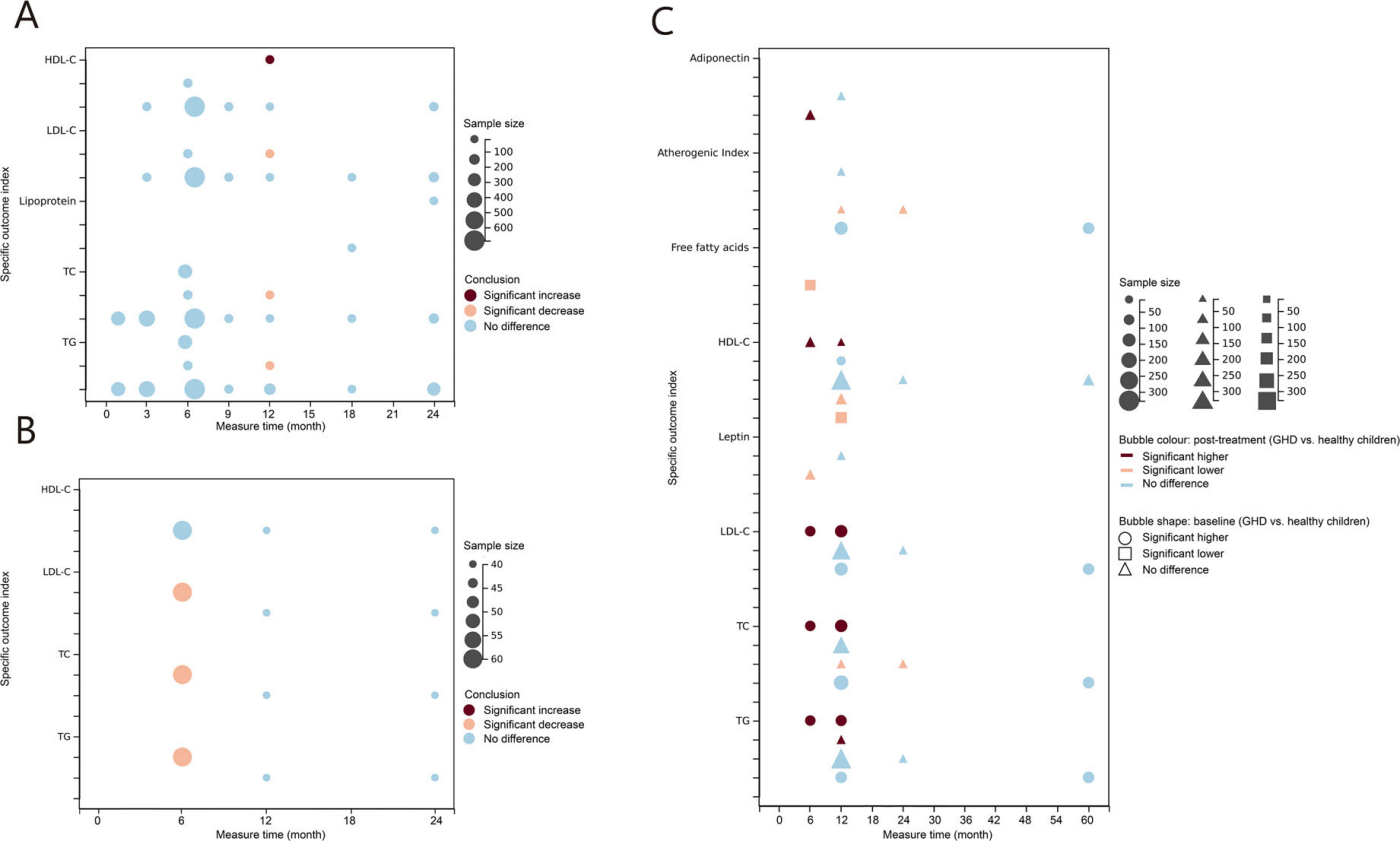
Evidence maps of the effect of GH on lipid metabolic outcomes. (A) Comparison before versus after GH treatment; (B) Comparison between GH treatment and untreated control; (C) Comparison between GHD and healthy children. The size of the bubble corresponds to the pooled sample size of studies with concordant effect direction at the given time point. The x‐axis denotes the follow‐up time in months. Abbreviations: GH, growth hormone; GHD, growth hormone deficiency; HDL‐C, high‐density lipoprotein cholesterol; LDL‐C, low‐density lipoprotein cholesterol; TC, total cholesterol; TG, triglyceride.

Twelve studies^11^, ^13^, ^17^, ^19^, ^20^, ^21^, ^24^, ^25^, ^28^, ^42^, ^43^, ^44^ (1635 participants) reported changes in lipid metabolic outcomes after GH treatment. Overall, GH therapy consistently showed NSI on lipoprotein levels. However, effects on high‐density lipoprotein cholesterol (HDL‐C), low‐density lipoprotein cholesterol (LDL‐C), total cholesterol (TC), and triglycerides (TG) were inconsistent. Most studies reported NSI on these outcomes; one study^17^ showed a significant increase in HDL‐C and significant decreases in LDL‐C, TC, and TG (Figure 3A).

Six studies^28^, ^31^, ^33^, ^34^, ^45^, ^46^ (209 participants) compared lipid metabolism in children with GHD according to GH treatment status. These studies showed that GH therapy did not significantly affect HDL‐C levels. Findings for LDL‐C and TC were mixed; equal numbers of studies revealed significant^33^, ^45^ and non‐significant^28^, ^31^ effects. Most studies^33^, ^45^, ^46^ showed a significant decrease in TG levels (Figure 3B).

Eleven studies^17^, ^36^, ^37^, ^38^, ^40^, ^41^, ^47^, ^48^, ^49^, ^50^, ^51^ (690 participants) compared lipid metabolism between children with GHD and healthy controls. However, the effects of GH treatment on lipid markers were inconsistent. Both significant and non‐significant effects were noted for the atherogenic index, LDL‐C, leptin, and TC; mixed findings were observed for adiponectin. Results concerning HDL‐C and TG were also inconsistent; one study^47^ showed a significant increase in TG and a significant decrease in HDL‐C after GH treatment (Figure 3C). One study reported NSI of GH on free fatty acids.^36^ The evidence map indicated that GH treatment did not significantly alter lipoprotein levels overall.

Meta‐analyses of seven studies^11^, ^15^, ^17^, ^19^, ^21^, ^42^, ^44^ revealed that medium‐dose GH yielded a significant reduction in TC of 0.32 mmol/L at follow‐up durations of ≥12 months, whereas high‐dose GH produced comparable decreases in TC (−0.34 mmol/L) and TG (−0.28 mmol/L) that did not exhibit statistical significance. Similarly, medium‐dose GH reduced LDL‐C by 0.33 mmol/L (not statistically significant), whereas high‐dose GH yielded a greater LDL‐C reduction (−0.64 mmol/L). Medium‐dose GH also produced a modest increase in HDL‐C of 0.20 mmol/L at ≥12 months; however, neither change displayed statistical significance (Figures S10–S16).

### Influence of GH treatment on calcium and phosphorus metabolic outcomes in children with GHD

**FIGURE 4:**
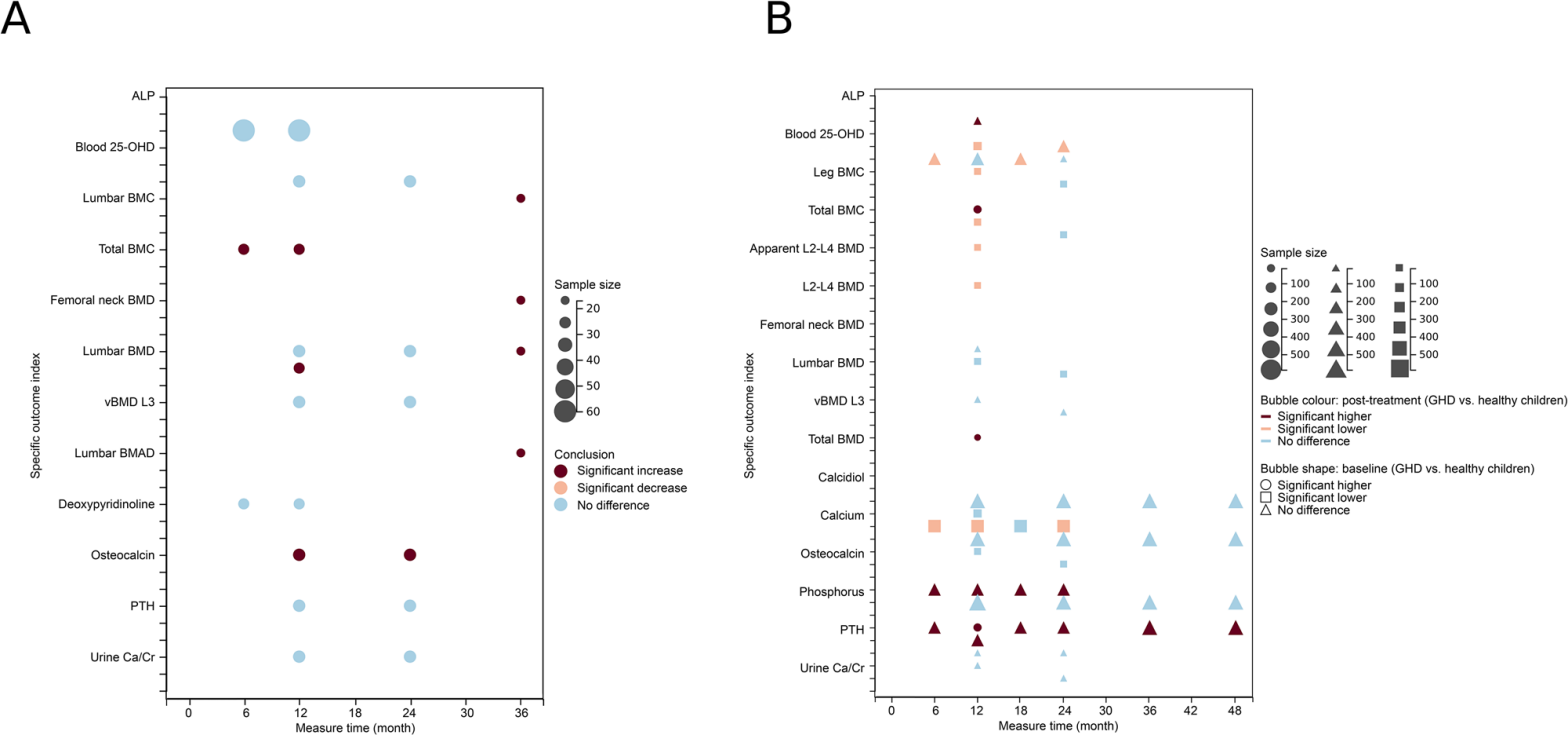
Evidence maps of the effect of GH on calcium and phosphorus metabolic outcomes. (A) Comparison before versus after GH treatment; (B) Comparison between GHD and healthy children. The size of the bubble corresponds to the pooled sample size of studies with concordant effect direction at the given time point. The x‐axis denotes the follow‐up time in months. Abbreviations: 25‐OHD, 25‐hydroxy vitamin D; ALP, alkaline phosphatase; BMAD, bone mineral apparent density; BMC, bone mineral content; BMD, bone mineral density; Ca/Cr, calcium and creatinine ratio; GH, growth hormone; GHD, growth hormone deficiency; PTH, parathyroid hormone; vBMD, volumetric bone mineral density.

Five studies^24^, ^27^, ^52^, ^53^, ^54^ (238 participants) examined changes in calcium and phosphorus metabolism after GH supplementation. Both significant and non‐siginificant impact were observed. Specifically, NSI was reported for alkaline phosphatase (ALP), serum 25‐hydroxyvitamin D (25‐OHD), volumetric bone mineral density at L3, deoxypyridinoline, parathyroid hormone (PTH), and the urinary calcium‐to‐creatinine ratio. In contrast, a significant increase was observed for femoral neck bone mineral density (BMD), total and lumbar bone mineral content (BMC), lumbar bone mineral apparent density, and osteocalcin. Findings for lumbar BMD were inconsistent; some studies showed an increase, others showed no effect (Figure 4A).

Two studies^28^, ^32^ (130 participants) compared calcium and phosphorus metabolic outcomes in children with GHD between those who received GH treatment and those who did not. Compared with untreated children, GH therapy showed no significant effect on BMD but produced a significant increase in serum 25‐OHD levels (Table S5).

Five studies^48^, ^55^, ^56^, ^57^, ^58^ (1157 participants) compared calcium and phosphorus metabolic outcomes between children with GHD after GH treatment and healthy controls. GH treatment significantly increased ALP and osteocalcin levels; it showed either NSI or an increase in BMC, BMD, serum calcium, phosphorus, and PTH levels. Additionally, GH treatment showed either NSI or a significant reduction in 25‐OHD levels, along with NSI on the urinary calcium‐to‐creatinine ratio (Figure 4B).

Overall, evidence mapping indicated that GH treatment may enhance bone turnover indices, such as osteocalcin. In contrast, its effects on mineral homeostasis and bone mass remain heterogeneous; serum calcium, phosphate, 25‐OHD, PTH, BMC, and BMD showed both significant and non‐significant effects.

Owing to the limited number of studies addressing individual mineral metabolism parameters, meta‐analytic synthesis was not feasible.

### Influence of GH treatment on thyroid hormone outcomes in children with GHD

**FIGURE 5:**
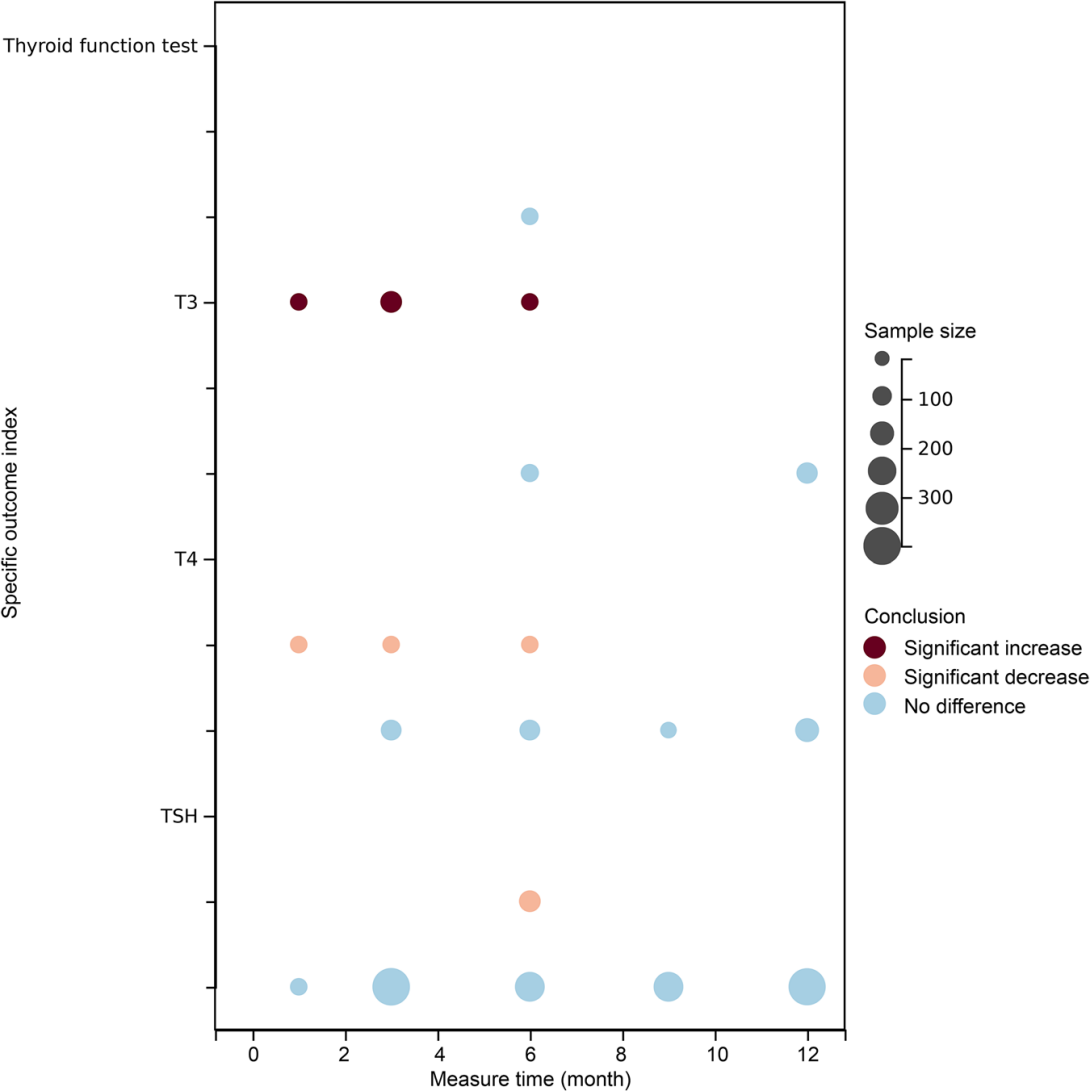
Evidence maps of the effect of GH on thyroid hormone outcomes before versus after GH treatment. The size of the bubble corresponds to the pooled sample size of studies with concordant effect direction at the given time point. The x‐axis denotes the follow‐up time in months. Abbreviations: GH, growth hormone; T3, triiodothyronine; T4, thyroxine; TSH, thyroid‐stimulating hormone.

Nine studies^12^, ^13^, ^14^, ^17^, ^18^, ^20^, ^22^, ^24^, ^59^ (654 participants) reported changes in thyroid hormone levels after GH supplementation. Overall, GH treatment showed NSI on thyroid function tests. However, effects on triiodothyronine (T3), thyroxine (T4), and thyroid‐stimulating hormone (TSH) levels were inconsistent. Most studies reported NSI, whereas a minority reported significant decreases in T4 and TSH levels. Two studies reported either increase^13^, ^22^ or no significant change^13^, ^18^ regarding T3 levels; one study^13^ reported an increase at 3 months but no change at 6 and 12 months (Figure 5).

Three studies^30^, ^31^, ^45^ (180 participants) compared thyroid hormone levels in children with GHD between those who received GH treatment and those who did not. GH therapy did not significantly affect free thyroxine (FT4), T3, or T4 levels. Results for free triiodothyronine (FT3) were inconsistent, whereas TSH levels either significantly decreased or remained unchanged (Table S6). No study compared thyroid hormone levels between children with GHD and healthy controls.

Evidence mapping demonstrated that GH treatment largely leaves the hypothalamic–pituitary–thyroid axis unchanged. TSH, T4, and FT4 most often remained stable; only occasional, transient reductions were observed in T4 and TSH. In contrast, FT3 and T3 showed the greatest variability (Figure 5 and Table S6).

Meta‐analysis of two studies^22^, ^45^ showed that low‐dose GH produced a trivial, non‐significant decrease in TSH of 0.05 mIU/L (95% confidence interval, −1.30 to 1.21). Insufficient reporting precluded meta‐analysis of additional thyroid parameters (Figure S17).

### Influence of GH treatment on body composition, WHR, and BMI in children with GHD

Several studies^13^, ^17^, ^28^, ^37^, ^43^, ^47^, ^48^, ^49^, ^52^, ^65^ examined the effects of GH treatment on body composition, WHR, and BMI in children with GHD (Table 1). Most studies indicated NSI concerning BMI after GH treatment, whereas two studies^13^, ^65^ reported a significant elevation. Three studies^28^, ^43^, ^48^ identified changes in body fat after GH treatment, although one study^43^ revealed no significant difference after adjustment for baseline overweight status. Two studies^28^, ^37^ examined WHR; one study^37^ noted that children with GHD had a higher WHR before treatment, which decreased to normal levels after GH therapy.

**TABLE 1:** Body composition, waist‐to‐hip ratio (WHR), and body mass index (BMI) reported in the included studies

Category of outcomes	Specific outcome	Total sample size	Study ID	Comparison	Measurement time (months)	Conclusion
Body composition	Body fat (%)	235	Stewart et al.^43^	Before vs. after GH treatment	NR	No significant difference
			Khadilkar et al.^48^	GHD vs. healthy children	12	Significantly lower
			Mauras et al.^28^	GH vs. no medical treatment	12/24	No significant difference^a^
	Fat mass	28	Zamboni et al.^52^	Before vs. after GH treatment	12/24	No significant difference
	Lean body mass	218	Mauras et al.^28^	GH vs. no medical treatment	12/24	No significant difference
			Khadilkar et al.^48^	GHD vs. healthy children	24	No significant difference
	Total/leg lean tissue mass	28	Zamboni et al.^52^	Before vs. after GH treatment	12/24	No significant difference
WHR	WHR	240	Capalbo et al.^37^	GHD vs. healthy children	12/60	Baseline: significantly higher Post‐treatment: no significant difference
			Mauras et al.^28^	Before vs. after GH treatment	24	No significant difference
BMI	BMI, SDS	669	Chen et al.^17^	Before vs. after GH treatment	12	No significant difference
			Du et al.^13^	Before vs. after GH treatment	3/6/12	Significant increase
			Rogol et al.^65^	Before vs. after GH treatment	36/48	Significant increase
			Zamboni et al.^52^	Before vs. after GH treatment	12/24	No significant difference
			Binay et al.^47^	GHD vs. healthy children	12	No significant difference^b^
			Capalbo et al.^49^	GHD vs. healthy children	24	No significant difference^b^
			Capalbo et al.^37^	GHD vs. healthy children	12/60	No significant difference^b^

^a^ Both groups increase, the mean change from basal to month 12 is significantly smaller in the GH‐treated group, with no significant difference at month 24.

^b^ Both baseline and post‐treatment.

Abbreviations: BMI, body mass index; GH, growth hormone; GHD, growth hormone deficiency; NR, not reported; SDS, standard deviation score; WHR, waist‐hip ratio.

### Influence of GH treatment on other outcomes in children with GHD

Studies summarized in Table 2 identified several emerging biomarkers. Monitoring of serum asymmetric dimethylarginine (ADMA) levels is clinically relevant, given that cardiovascular and renal diseases often originate in childhood. Önder et al.^71^ found that GH treatment increased ADMA levels. Zeng and Hu^32^ documented lower gastrin levels in children with GHD and noted that GH therapy increased those levels. Esposito et al.^70^ showed that, although GHD impairs erythropoiesis, GH therapy improves red blood cell production.

**TABLE 2:** Other biomarkers identified in the included studies

Study ID	Objective of the tested biomarkers	Other biomarkers	Comparison	Time point (month)	Conclusion
Önder et al.^71^	Cardiovascular and renal system disorder	Serum asymmetric dimethylarginine level	GHD vs. healthy children	3/6	Baseline: no significant difference; post‐treatment: significantly higher
Zeng et al.^32^	Safety	Gastrin	GH vs. no medical treatment	12	Significant increase
Esposito et al.^70^	Erythropoiesis	Hemoglobin	GHD vs. healthy children	12/24	Significantly lower^a^
				36/48/60	Baseline: significantly lower; post‐treatment: no significant difference
		Red cells	GHD vs. healthy children	12	Significantly lower^a^
				24/36/48/60	Baseline: significantly lower; post‐treatment: no significant difference
		Hematocrit	GHD vs. healthy children	12	Significantly lower^a^
				24/36/48/60	Baseline: significantly lower; post‐treatment: no significant difference
		MCV, MCH, and RDW	GHD vs. healthy children	12/24/36/48/60	No significant difference^a^
	Leucopoiesis	Leukocytes, neutrophils lymphocytes, monocytes	GHD vs. healthy children	12/24/36/48/60	No significant difference^a^
	Thrombopoiesis	Platelets	GHD vs. healthy children	12/24/36/48/60	No significant difference^a^
Andiran and Yordam^72^	Modulate the release of some cytokines	Tumor necrosis factor	Before vs. after GH treatment	6/12	Significant decrease
			GHD vs. healthy children	6/12	Baseline: significantly higher; Post‐treatment: No report
Sinués et al.^73^	CYP3A activity	6β‐hydroxycortisol/ free cortisol ratio	GHD vs. healthy children	1	Total and female group: significantly higher^a^
					Male: baseline, significantly higher; post‐treatment, no significant difference
Neyzi et al.^67^	Liver function	ALT and AST	Before vs. after GH treatment	3/6	Significant increase
		Serum urea levels	Before vs. after GH treatment	3/6	Significant decrease
Wang et al.^62^	Endogenous ligand of the GH secretin receptor	Serum ghrelin level	Before vs. after GH treatment	3/6	PGHD group: significant decrease
					CGHD group: significant increase
			GHD vs. healthy children	3	PGHD group: significantly higher^a^
					CGHD group: significantly lower^a^
				6	PGHD group: baseline, significantly higher; post‐treatment, significantly lower
					CGHD group: significantly lower^a^
	Safety	Serum nesfatin‐1 level	Before vs. after GH treatment	3/6	PGHD group: significant decrease
					CGHD group: significant decrease
			GHD vs. healthy children	3/6	PGHD group: significantly higher^a^
					CGHD group: significantly higher^a^
Zamboni et al.^52^	Bone mineralization	N‐terminal telopeptide of type I collagen levels	Before vs. after GH treatment	12/24	No significant difference
			GHD vs. healthy children	12	Significantly higher^a^
Zelinska et al.^18^	Safety	Cortisol levels	Before vs. after GH treatment	12	No significant difference
Calzada‐León et al.^20^	Biochemical safety	Blood cytometry, blood chemistry, electrolytes, hepatic profiles, and urine analysis	Before vs. after GH treatment	Every 2	No significant difference

^a^ Both baseline and post‐treatment.

Abbreviations: ALT, alanine aminotransferase; AST, aspartate aminotransferase; CGHD, complete absence of growth hormone; GH, growth hormone; GHD, growth hormone deficiency; MCV, mean corpuscular volume; MCH, mean corpuscular hemoglobin; PGHD, partial absence of growth hormone; RDW, red cell distribution width.

Andiran and Yordam^72^ reported that GH exerts an inhibitory effect on tumor necrosis factor. Cytochrome P450 3A (CYP3A) influences the metabolism of steroids and other drugs. CYP3A activity is higher in children with GHD than in those without GHD, regardless of sex. GH treatment for 30 days normalized CYP3A activity in boys, whereas no significant change was observed in girls.^73^ GH therapy also affected liver‐related parameters, including alanine aminotransferase, aspartate aminotransferase, and urea levels. Neyzi et al.^67^ found that alanine aminotransferase and aspartate aminotransferase levels remained within the normal range but slightly increased in GH‐treated children with GHD, whereas urea levels decreased.

Nesfatin‐1 and ghrelin influence the hypothalamic–pituitary–gonadal axis, energy expenditure, appetite, and insulin secretion; they are thought to be affected by GHD. Wang et al.^62^ found that before GH treatment, ghrelin levels were higher in patients with partial GHD and lower in patients with complete GHD than in healthy controls. Nesfatin‐1 levels were higher in both patient groups than in healthy individuals. After 3 and 6 months of treatment, ghrelin and nesfatin‐1 levels both decreased in the partial GHD group, whereas ghrelin levels increased and nesfatin‐1 levels decreased in the complete GHD group.

N‐terminal telopeptide of type I collagen (NTx) is a marker of bone resorption. Zamboni et al.^52^ found that urinary NTx levels were higher in patients with GHD than in healthy individuals at baseline and after 1 year of GH treatment. Although NTx levels decreased after 1 year of GH therapy and increased again after 2 years, these changes were not statistically significant. Esposito et al.^70^ reported that GH treatment did not affect leukocyte or platelet counts. Zelinska et al.^18^ and Calzada‐León et al.^20^ showed that GH therapy did not alter cortisol levels, routine blood test results, electrolyte levels, liver function, or urinalysis findings.

## DISCUSSION

This evidence map systematically summarizes current data regarding GH therapy in children with GHD. It also addresses gaps identified in the 2016 Pediatric Endocrine Society and 2019 Growth Hormone Research Society guidelines by incorporating metabolic outcomes (e.g., body composition) and emerging biomarkers for diagnosis and treatment. However, definitions of GHD varied across studies, and nutritional status was often not reported in pediatric populations. Additionally, GH dose, formulation, and route of administration differed; treatment duration in most studies was ≤12 months.

Regarding the effects of GH treatment on glucose metabolism in children with GHD, most studies reported NSI on AUCglu, insulin sensitivity, or postprandial glucose levels. Several studies indicated that GH significantly increased fasting insulin, fasting plasma glucose, and HbA1c levels; most others reported NSI concerning these indicators. Findings from systematic reviews and meta‐analyses suggest that GH produces only modest increases in fasting glucose and HbA1c, such that values remain within the normal range. Fasting insulin levels are also affected to a limited extent, although higher doses and longer follow‐up durations may intensify this effect. Data regarding outcomes such as postprandial glucose, AUCglu, and insulin sensitivity remain limited. Moreover, long‐term evidence beyond 12 months, particularly for >24 months, is scarce. Few studies have compared GH treatment and no treatment with respect to glucose metabolic outcomes.

The effects of rhGH on HDL‐C, LDL‐C, TC, and TG were variable. Most studies showed NSI concerning these parameters, whereas a minority reported either significant increases or decreases in specific indicators. Systematic reviews and meta‐analyses revealed that GH significantly reduced TC with longer follow‐up; it may decrease TG and LDL‐C, while increasing HDL‐C. However, these latter effects did not exhibit statistical significance. Data remain limited for several lipid‐related outcomes, including adiponectin, atherogenic index, free fatty acids, leptin, and lipoproteins. Furthermore, long‐term follow‐up data beyond 12 months, particularly for >24 months, are lacking. Most available results were obtained within 24 months; only one study reported outcomes at 60 months. Few studies have compared GH treatment versus no treatment or evaluated differences between children with GHD and healthy controls.

Available evidence indicates that rhGH exerts heterogeneous effects on mineral homeostasis and bone mass, and it may enhance certain bone turnover indices. Data remain insufficient regarding calcium and phosphorus outcomes with follow‐up periods exceeding 24 months.

To date, no studies have compared thyroid hormone levels between children with GHD who receive treatment and healthy controls. Additionally, the effects of GH on T3, T4, and TSH levels remain inconclusive, given that only slight changes in T4 and TSH were reported in a few studies. Our meta‐analysis result indicated a non‐significant reduction in TSH after GH treatment; however, this finding was only based on two studies. Additional primary research is required to clarify these associations. Moreover, studies assessing thyroid hormone outcomes beyond 12 months are lacking, and data quantifying long‐term TSH changes remain limited. Beyond thyroid outcomes, further investigation is needed to determine the effects of GH treatment on calcium and phosphorus metabolism across the three comparison types.

Integration of available evidence suggests that GH influences fat and mineral metabolism by promoting lipolysis and inhibiting lipogenesis, as well as by affecting the absorption and utilization of minerals (e.g., calcium and phosphorus).

A systematic review^74^ demonstrated that GH therapy in children with GHD reduced fat mass while increasing lean body mass, underscoring the importance of periodically assessing body composition. Another review^75^ comparing long‐acting GH with daily GH reported similar efficacy, safety, adherence, and quality of life. Additionally, a meta‐analysis^76^ of 16 trials (*n* = 1319) showed that rhGH therapy in idiopathic GHD significantly reduced TC and increased HDL‐C, suggesting favorable lipid metabolic effects that depend on treatment duration.

In contrast, the present study incorporated a broader evidence base (39 RCTs and 24 observational studies published in either English or Chinese) and examined the metabolic effects of GH across three comparison types. Most studies reported no significant changes in lipid metabolism; only modest improvements in HDL‐C and TC were noted, which differs from the findings of Yuan et al.’s^76^ study restricted to clinical trials reported in the English language (16 studies). Furthermore, we expanded metabolic outcome assessments to include glucose metabolism, calcium and phosphorus metabolism, thyroid hormone profiles, WHR, and novel biomarkers; prior reviews focused on additional endpoints, such as cost‐effectiveness and quality of life.

Our study has some important strengths and limitations. The strengths include (i) its use of a systematic literature search to minimize selection bias and (ii) its comprehensive evaluation of the effects of GH treatment on metabolic outcomes. This approach provides a robust evidence base to support clinical decision‐making and guideline development.

The limitations include insufficient data to assess differences regarding the effects of GH supplementation between adolescents and children according to nutritional status. Additionally, heterogeneity in GHD definitions, age groups, baseline glucose and lipid levels, GH doses, and treatment durations contributed to inconsistent findings. Given that most studies were limited to 12 months of follow‐up and data beyond 24 months were sparse, the present findings cannot clarify whether lipid‐related improvements persist into adolescence or adulthood, nor can they exclude the potential for late adverse effects. Our findings exclusively apply to pediatric patients with GHD. The effects of GH therapy in non‐GHD populations, including individuals with Turner syndrome or short‐stature homeobox‐containing (SHOX) gene alterations, were not evaluated and may differ from effects observed in this cohort.

In conclusion, the findings of this review indicate that the effects of GH treatment on glucose and thyroid hormone levels are consistent with clinical observations; this treatment may have favorable effects on lipid metabolism. Evidence regarding impacts on body composition, BMI, and WHR remains limited, underscoring the need for further investigation. Future clinical studies should address specific pediatric age groups, variations in GHD severity, multiple nutritional statuses, diverse GH doses and formulations, and extended follow‐up durations.

## CONFLICT OF INTEREST

Wei Wu, Ningyi Song, Yue Zhao, and Caiqi Du declare no conflict of interest. Yuning Zhao and You Wu are employees and stockholders of Novo Nordisk, but are not involved in data processing. Xiaoping Luo serves as an advisor to GenSci, Amoytop, Novo Nordisk, Takeda, Lumos, Sanofi, Medtronic, Ipsen, Visen, and Kyowa Kirin, providing research support for GenSci, Amoytop, Novo Nordisk, Takeda, Lumos, Sanofi, Medtronic, Ipsen, Visen, and Kyowa Kirin, and is a speaker for GenSci, Novo Nordisk, and Visen. Xiaoping Luo is a member of the *Pediatric Investigation* Editorial Board. The study design, analytical plan, and interpretation remained under the full control of the academic authors, and the outcome selection was driven by clinical relevance and protocol pre‐specification.

## Supporting information



Supporting Information
