# Supplementary material for: Evidence map and gap analysis of metabolic change in pediatric growth hormone deficiency treated with growth hormone
Source: Pediatr Investig. 2026 Mar 11;10(4):359–73. doi: 10.1002/ped4.70050 (PMC13398671; doi:10.1002/ped4.70050)
Supplement: Supplementary file 1 [file PED4-10-359-s001.pdf]

Supplementary Material for

Evidence map and gap analysis of metabolic change in pediatric growth hormone deficiency treated with growth hormone

Wei Wu, Ningyi Song, Yue Zhao, Caiqi Du, Yuning Zhao, You Wu, Xiaoping Luo

Table S1. Search strategy in PubMed, EMBASE, Cochrane Library, China National Knowledge Infrastructure (CNKI), Wanfang, and Chinese Biomedical Literature Database (CBM)

**PubMed**		
**No.**	**Search terms**	**No. of records**
#1	"Dwarfism, Pituitary"[Mesh] OR "growth hormone deficienc*"[tw] OR GHD[tw] OR "Pituitary Dwarf*"[tw] OR "Hyposomatotrophic Dwarf*"[tw] OR "Pituitary Nanism*"[tw] OR "HGH Deficienc*"[tw] OR "Somatotropin Deficienc*"[tw] OR "GH Deficienc*"[tw] OR "Hypophysial Dwarf*"[tw]	—
#2	"Human Growth Hormone"[Mesh] OR "Human Growth Hormon*"[tw] OR Somatotropin*[tw] OR Somatropin[tw] OR hGH[tw] OR accretropin[tw] OR "bio tropin"[tw] OR "genotropin miniquick"[tw] OR jintropin[tw] OR "lb 03002"[tw] OR norditropin[tw] OR nutropin[tw] OR nutropinaq[tw] OR omnitrope[tw] OR saizen[tw] OR "tev tropin"[tw] OR tevtropin[tw] OR valtropin[tw] OR Somatropin[tw] OR Serostim[tw] OR Zomacton[tw] OR "Cryo Tropin"[tw] OR CryoTropin[tw] OR "r-Hgh"[tw] OR rhGH[tw] OR "r-gh"[tw] OR rGH[tw] OR Humatrope[tw] OR Umatrope[tw] OR Nutropin[tw] OR Genotropin[tw] OR Genotonorm[tw]	—
#3	"Child"[Mesh] OR "Infant"[Mesh] OR "Adolescent"[Mesh] OR child[tw] OR children[tw] OR Infant[tw] OR infants[tw] OR infancy[tw] OR newborn[tw] OR newborns[tw] OR "new-born"[tw] OR "new-borns"[tw] OR neonate[tw] OR neonates[tw] OR neonatal[tw] OR "neo-nate"[tw] OR "neo-nates"[tw] OR "neo-natal"[tw] OR neonatology[tw] OR NICU[tw] OR premature[tw] OR prematures[tw] OR "pre-mature"[tw] OR "pre-matures"[tw] OR preterm[tw] OR "pre-term"[tw] OR postnatal[tw] OR "post-natal"[tw] OR baby[tw] OR babies[tw] OR suckling[tw] OR sucklings[tw] OR toddler[tw] OR toddlers[tw] OR childhood[tw] OR schoolchild[tw] OR schoolchildren[tw] OR childcare[tw] OR "child-care"[tw] OR preschool[tw] OR "pre-school"[tw] OR kid[tw] OR kids[tw] OR boy[tw] OR boys[tw] OR girl[tw] OR girls[tw] OR Pediatrics[tw] OR Pediatric[tw] OR Pediatrics[tw] OR Paediatric[tw] OR Paediatrics[tw] OR PICU[tw] OR Adolescen*[tw] OR Teen*[tw] OR Youth*[tw] OR juvenile[tw]	—
#4	"Meta-Analysis"[pt] OR "Meta-Analysis as Topic"[Mesh] OR "Systematic Review" [pt] OR "Systematic Reviews as Topic"[Mesh] OR "systematic"[Filter] OR "Systematic Review*"[tiab] OR "Meta-Analysis"[tiab] OR Metaanalys*[tiab]	—
#5	("controlled clinical trial"[pt] OR "Controlled Clinical Trials as Topic"[MeSH] OR "Random Allocation"[MeSH] OR "Double-Blind Method"[MeSH] OR "single-blind method"[MeSH] OR "Control Groups"[MeSH] OR "cross-over studies"[MeSH] OR random*[tiab] OR placebo[tiab] OR trial[tiab] OR groups[tiab] OR crossover[tiab] OR cross-over[tiab]) NOT ("Animals"[Mesh] NOT ("Humans"[Mesh] AND "Animals"[Mesh]))	—
#6	("Observational Study"[pt] OR "Observational Studies as Topic"[Mesh] OR "Cohort Studies"[Mesh] OR "Case-Control Studies"[Mesh] OR "Cross-Sectional Studies"[Mesh] OR Observational Stud*[tiab] OR Cohort[tiab] OR "Follow-Up"[tiab] OR Longitudinal*[tiab] OR Prospectiv*[tiab] OR Retrospectiv*[tiab] OR "Case-Control"[tiab] OR "Cross-Sectional"[tiab]	—

	OR "case series"[tiab] OR "single arm"[tiab]) NOT ("Animals"[Mesh] NOT ("Humans"[Mesh] AND "Animals"[Mesh]))	
#7	#1 and #2	5728
#8	#1 and #2 and #3	3783
#9	#8 and #4	29
#10	#8 and #5	878
#11	#8 and #6	1182
**EMBASE**		
**No.**	**Search terms**	**No. of records**
#1	'pituitary dwarfism'/exp OR (GHD OR (("growth hormone" OR GH OR Somatotropin OR HGH) NEAR/3 deficienc*) OR ((Pituitary OR Hyposomatotrophic OR Hypophysial) NEAR/3 Dwarf*) OR "Pituitary Nanism*"):ab,ti,kw	—
#2	'human growth hormone'/exp OR ("Human Growth Hormon*" OR Somatotropin* OR Somatropin OR hGH OR accretropin OR "bio tropin" OR "genotropin miniquick" OR jintropin OR "lb 03002" OR norditropin OR nutropin OR nutropinaq OR omnitrope OR saizen OR "tev tropin" OR tevtropin OR valtropin OR Somatropin OR Serostim OR Zomacton OR "Cryo Tropin" OR CryoTropin OR "r-Hgh" OR rhGH OR "r-gh" OR rGH OR Humatrope OR Umatrope OR Nutropin OR Genotropin OR Genotonorm):ab,ti,kw	—
#3	'adolescent'/exp OR 'child'/exp OR (child OR children OR Infant OR infants OR infancy OR newborn OR newborns OR "new-born" OR "new-borns" OR neonate OR neonates OR neonatal OR "neo-nate" OR "neo-nates" OR "neo-natal" OR neonatology OR NICU OR premature OR prematures OR "pre-mature" OR "pre-matures" OR preterm OR "pre-term" OR postnatal OR "post-natal" OR baby OR babies OR suckling OR sucklings OR toddler OR toddlers OR childhood OR schoolchild OR schoolchildren OR childcare OR "child-care" OR preschool OR "pre-school" OR kid OR kids OR boy OR boys OR girl OR girls OR Pediatrics OR Pediatric OR Pediatrics OR Paediatric OR Paediatrics OR PICU OR Adolescen* OR Teen* OR Youth* OR juvenile):ab,ti,kw	—
#4	'meta analysis'/exp OR 'meta analysis (topic)'/exp OR 'systematic review'/exp OR 'systematic review (topic)'/exp OR (Systemat* NEAR/3 Review* OR 'Meta Analysis*' OR Metaanalys*):ab,ti,kw	—
#5	('controlled clinical trial'/exp OR 'Controlled Clinical Trial (Topic)'/exp OR 'double blind procedure'/de OR 'control group'/de OR 'crossover procedure'/de OR 'single blind procedure'/de OR 'triple blind procedure'/de OR 'placebo'/de OR 'randomization'/exp OR (random* OR trial OR groups OR placebo* OR crossover OR "cross-over"):ab,ti,kw) AND 'human'/exp	—

#6	('observational study'/exp OR 'cohort analysis'/exp OR 'cross-sectional study'/exp OR 'case control study'/exp OR ("observational stud*" OR Cohort OR "Follow-Up" OR Longitudinal* OR Prospectiv* OR Retrospectiv* OR "Case-Control" OR "Cross-Sectional" OR "case-series" OR "single arm"):ab,ti,kw) AND 'human'/exp	—
#7	#1 and #2	3704
#8	#1 and #2 and #3	2555
#9	#8 and #4	30
#10	#8 and #5	695
#11	#8 and #6	628
**The Cochrane Library**		
**No.**	**Search terms**	**No. of records**
#1	MeSH descriptor: [Dwarfism, Pituitary] explode all trees	—
#2	(GHD OR (("growth hormone" OR GH OR Somatotropin OR HGH) NEAR/3 deficienc*) OR ((Pituitary OR Hyposomatotrophic OR Hypophysial) NEAR/3 Dwarf*) OR "Pituitary Nanism*"):ti,ab,kw	—
#3	MeSH descriptor: [Human Growth Hormone] explode all trees	—
#4	("Human Growth Hormon*" OR Somatotropin* OR Somatropin OR hGH OR accretropin OR "bio tropin" OR "genotropin miniquick" OR jintropin OR "lb 03002" OR norditropin OR nutropin OR nutropinaq OR omnitrope OR saizen OR "tev tropin" OR tevtropin OR valtropin OR Somatropin OR Serostim OR Zomacton OR "Cryo Tropin" OR CryoTropin OR "r-Hgh" OR rhGH OR "r-gh" OR rGH OR Humatrope OR Umatrope OR Nutropin OR Genotropin OR Genotonorm):ti,ab,k	—
#5	MeSH descriptor: [Child] explode all trees	—
#6	MeSH descriptor: [Infant] explode all trees	—
#7	MeSH descriptor: [Adolescent] explode all trees	—
#8	(child OR children OR Infant OR infants OR infancy OR newborn OR newborns OR "new-born" OR "new-borns" OR neonate OR neonates OR neonatal OR "neo-nate" OR "neo-nates" OR "neo-	—

	natal" OR neonatology OR NICU OR premature OR prematures OR "pre-mature" OR "pre-matures" OR preterm OR "pre-term" OR postnatal OR "post-natal" OR baby OR babies OR suckling OR sucklings OR toddler OR toddlers OR childhood OR schoolchild OR schoolchildren OR childcare OR "child-care" OR preschool OR "pre-school" OR kid OR kids OR boy OR boys OR girl OR girls OR Pediatrics OR Pediatric OR Pediatrics OR Paediatric OR Paediatrics OR PICU OR Adolescen* OR Teen* OR Youth* OR juvenile):ti,ab,kw	
#9	#1 or #2	1225
#10	#3 or #4	1383
#11	#9 and #10	745
#12	#5 or #6 or #7 or #8	316819
#13	#11 and #12	420 (419 trials, one review)
**CNKI** (期刊、学位、会议，中英文扩展：是，中文)		
**No.**	**Search terms**	**No. of records**
#1	(SU%=垂体性侏儒症+垂体性侏儒+垂体侏儒症+生长激素缺乏症+生长激素缺乏 OR TKA=垂体性侏儒症+垂体性侏儒+垂体侏儒症+生长激素缺乏症+生长激素缺乏) AND (SU%=重组人生长激素+人生长激素+人重组生长激素+重组生长激素+ hGH+rhGH+ rGH+ "r-hGH"+ "r-GH" OR TKA=重组人生长激素+人生长激素+人重组生长激素+重组生长激素+ hGH +rhGH+ rGH+ "r-hGH"+ "r-GH") AND (SU%=儿童+婴儿+幼儿+婴幼儿+少儿+小儿+学龄+学龄前+学生+小学生+早产儿+新生儿+儿科+低龄+适龄+患儿+学龄+小学生+中学生+学生+青少年+少年 OR TKA=儿童+婴儿+幼儿+婴幼儿+少儿+小儿+学龄+学龄前+学生+小学生+早产儿+新生儿+儿科+低龄+适龄+患儿+学龄+小学生+中学生+学生+青少年+少年)	—
#2	(SU%=META+荟萃+系统综述+系统评价+评价综述 OR TKA=META+荟萃+系统综述+系统评价+评价综述)	—
#3	(SU%=随机+盲法+双盲+单盲+三盲+交叉+RCT OR TKA=随机+盲法+双盲+单盲+三盲+交叉+RCT)	—
#4	(TKA=观察性研究+病例对照+队列+横断面+纵向+前瞻+回顾+追踪调查+调查追踪+跟踪调查+随访 OR TI=病例+观察 OR SU%=观察性研究+病例对照+队列+横断面+纵向+前瞻+回顾+追踪调查+跟踪调查+随访)	—

#5	#1 and #2	7
#6	#1 and #3	69
#7	#1 and #4	208
**Wanfang**		
**No.**	**Search terms**	**No. of records**
#1	主题:("垂体性侏儒症" OR "垂体性侏儒" OR "垂体侏儒症" OR "生长激素缺乏症" OR "生长激素缺乏") and 主题:("人生长激素" OR "人重组生长激素" OR "重组生长激素" OR hGH OR rhGH OR rGH OR "r-hGH" OR "r-GH") and 主题:(儿童 OR 婴儿 OR 幼儿 OR 婴幼儿 OR 少儿 OR 小儿 OR 学龄 OR 学龄前 OR 学生 OR 小学生 OR 早产儿 OR 新生儿 OR 儿科 OR 低龄 OR 适龄 OR 患儿 OR 学龄 OR 小学生 OR 中学生 OR 学生 OR 青少年 OR 少年)	—
#2	主题:(META OR "荟萃" OR "系统综述" OR "系统评价" OR "评价综述")	—
#3	主题:("随机" OR "盲法" OR "双盲" OR "单盲" OR "三盲" OR "交叉" OR "RCT")	—
#4	主题:("观察性研究" OR "病例对照" OR "队列" OR "横断面" OR "纵向" OR "前瞻" OR "回顾" OR "追踪调查" OR "调查追踪" OR "跟踪调查" OR "随访") or 题名:("病例" OR "观察")	—
#5	#1 and #2	3
#6	#1 and #3	26
#7	#1 and #4	118
**CBM**		
**No.**	**Search terms**	**No. of records**
#1	("侏儒症，垂体性"[不加权:扩展] OR "垂体性侏儒症"[常用字段:智能] OR "垂体性侏儒"[常用字段:智能] OR "垂体侏儒症"[常用字段:智能] OR "生长激素缺乏症"[常用字段:智能] OR "生长激素缺乏"[常用字段:智能]) AND ("人生长激素"[不加权:扩展] OR "重组人生长激素"[常用字段:智能] OR "人生长激素"[常用字段:智能] OR "人重组生长激素"[常用字段:智能] OR "重组生长激素"[常用字段:智能] OR "hGH"[常用字段:智能] OR "rhGH"[常用字段:智能]	—

	OR "rGH"[常用字段:智能] OR "r-hGH"[常用字段:智能] OR "r-GH"[常用字段:智能]) AND ("儿童"[常用字段:智能] OR "婴儿"[常用字段:智能] OR "幼儿"[常用字段:智能] OR "婴幼儿"[常用字段:智能] OR "少儿"[常用字段:智能] OR "小儿"[常用字段:智能] OR "学龄"[常用字段:智能] OR "学龄前"[常用字段:智能] OR "学生"[常用字段:智能] OR "小学生"[常用字段:智能] OR "早产儿"[常用字段:智能] OR "新生儿"[常用字段:智能] OR "儿科"[常用字段:智能] OR "低龄"[常用字段:智能] OR "适龄"[常用字段:智能] OR "患儿"[常用字段:智能] OR "学龄"[常用字段:智能] OR "小学生"[常用字段:智能] OR "中学生"[常用字段:智能] OR "学生"[常用字段:智能] OR "青少年"[常用字段:智能] OR "少年"[常用字段:智能])	
#2	("Meta 分析"[不加权:扩展] OR "Meta 分析(主题)"[不加权:扩展] OR "META"[常用字段:智能] OR "荟萃"[常用字段:智能] OR "系统综述"[常用字段:智能] OR "系统评价"[常用字段:智能] OR "评价综述"[常用字段:智能])	—
#3	("临床对照试验"[不加权:扩展] OR "临床对照试验(主题)"[不加权:扩展] OR "随机对照试验"[不加权:扩展] OR "随机对照试验(主题)"[不加权:扩展] OR "非随机对照试验(主题)"[不加权:扩展] OR "随机"[常用字段:智能] OR "盲法"[常用字段:智能] OR "双盲"[常用字段:智能] OR "单盲"[常用字段:智能] OR "三盲"[常用字段:智能] OR "交叉"[常用字段:智能] OR "RCT"[常用字段:智能])	—
#4	("观察性研究"[不加权:扩展] OR "观察性研究(主题)"[不加权:扩展] OR "队列研究"[不加权:扩展] OR "横断面研究"[不加权:扩展] OR "观察性研究"[常用字段:智能] OR "病例对照"[常用字段:智能] OR "队列"[常用字段:智能] OR "横断面"[常用字段:智能] OR "纵向"[常用字段:智能] OR "前瞻"[常用字段:智能] OR "回顾"[常用字段:智能] OR "追踪调查"[常用字段:智能] OR "调查追踪"[常用字段:智能] OR "跟踪调查"[常用字段:智能] OR "随访"[常用字段:智能] OR "病例"[中文标题:智能] OR "观察"[中文标题:智能])	—
#5	#1 and #2	4
#6	#1 and #3	33
#7	#1 and #4	169

Six databases were searched from the inception to 25th of July, 2023, including PubMed, EMBASE, Cochrane Library, China National Knowledge Infrastructure (CNKI), Wanfang and China Biology Medicine disc (CBM).

Table S2. Baseline characteristics of populations in the included studies

Value	Total (*n* = 6158)		Before and after GH supplement (*n*= 3409)		GH supplement compared with non-medical treatment (*n* = 645)		Compared with healthy population (*n* = 2550)	
	No. of studies	Sample size	No. of studies	Sample size	No. of studies	Sample size	No. of studies	Sample size
Age (years)								
3–≤9	5	562 (9.1)	3	127 (3.7)	1	90 (14.0)	2	395 (15.5)
9–≤12	0	0	0	0	0	0	0	0
12–≤18	1	17 (0.3)	1	17 (0.5)	0	0	0	0
Mixed	53	4753 (77.2)	28	2481 (72.8)	9	513 (79.5)	17	1798 (70.5)
NR	4	826 (13.4)	3	784 (23.0)	1	42 (6.5)	4	357 (14.0)

Adolescent developmental state								
Prepubertal	48	5351 (86.9)	27	3119 (91.5)	5	295 (45.7)	19	2215 (86.9)
During	10	345 (5.6)	5	163 (4.8)	2	82 (12.7)	4	158 (6.2)
After puberty	0	0	0	0	0	0	0	0
NR	9	462 (7.5)	3	127 (3.7)	5	268 (41.6)	3	177 (6.9)
Peak GH (ng/mL)								
<5 ng/mL	6	836 (13.6)	5	776 (22.8)	1	60 (9.3)	0	0
<7 ng/mL	4	166 (2.7)	1	17 (0.5)	2	49 (7.6)	1	100 (3.9)
<10 ng/mL	14	1149 (18.7)	6	370 (10.8)	2	142 (22.0)	5	474 (18.6)
Mixed	24	2497 (40.5)	16	1913 (56.1)	3	210 (32.6)	8	587 (23.0)
NR	15	1510 (24.5)	7	333 (9.8)	3	184 (28.9)	9	1389 (54.5)

Nourishment status								
Good	3	304 (5.0)	3	304 (8.9)	0	0	0	0
NR	60	5854 (95.0)	32	3105 (91.1)	11	645 (100.0)	23	2550 (100.0)
Causes of* GHD								
Isolated GHD	35	1599 (26.0)	16	806 (23.6)	5	249 (38.6)	18	747 (29.3)
MPHD	11	224 (3.6)	6	174 (5.1)	1	11 (1.7)	5	50 (2.0)
Intracranial causes of GHD	7	88 (1.4)	3	55 (1.6)	3	28 (4.3)	1	5 (0.2)
Other type of GHD	13	326 (5.3)	8	262 (7.7)	3	18	3	57 (2.2)
Unknown	28	2426 (39.4)	22	1984 (58.2)	6	339	5	196 (7.7)
NA	23	1495 (24.3)	3	128 (3.8)	0	0	23	1495 (58.6)

GH, growth hormone; GHD, growth hormone deficiency; MPHD, Multiple Pituitary Hormone Deficiency; NA, not applicable; NR, not reported.

Table S3. Summary of detection methods for metabolic outcome-related indicators specifically reported in the included studies

Metabolic outcome	Detection method	Specific indicators	Number of studies	Sample size
Glucose metabolism	Glucose oxidase method	None	6	384
	ELISA	None	3	94
	HPLC	HbA1c, Insulin	2	140
	CLIA	Insulin	1	36
	Hexokinase method	None	1	58
	Solid-phase radioimmunoassay	Insulin	1	24
Lipid metabolism	Enzymatic method	None	5	329
	Biochemical enzymatic colorimetry	None	3	118
	ELISA	Adiponection, leptin	2	88
Calcium and phosphorus metabolism	RIA	25 (OH)D3, PTH	2	107
	DXA	BMD, BMC	2	126
	Colorimetric Methods	Calsium and Phosphorus plasma level	2	583
	CLIA	PTH	1	98
	HPLC	25-OHD, urinary phosphate excretion	1	28

Thyroid level	ELISA	None	2	162
	Automated chemiluminescence system (Immulite analyzer)	None	1	487
	ECL Assay	FT4, FT3, T4, T3, TSH,	1	60

25 (OH)D3, 25-hydroxyvitamin D; ALT, alanine aminotransferase; AST, aspartate aminotransferase; BMC, bone mineral content; BMD, bone mineral density; CLIA, chemiluminescence immunoassays; DXA, dual-energy X-ray absorptiometry; ECL, Electrochemiluminescence Assay; ELSA, enzyme-linked immunosorbent assay; FT3, free triiodothyronine; FT4, free thyroxine; HbA1c, hemoglobin A1c; HPLC, high-performance liquid chromatography; PTH, parathyroid hormone; RIA, radioimmunoassay; T3, triiodothyronine; T4, thyroxine; TSH, thyroid-stimulating hormone;

Table S4. Glucose metabolic outcomes between GH treatment and non-medical treatment in GHD children

Specific outcomes	No. of studies	Study ID	Measurement time (month)	Conclusion
**Fasting plasma glucose**	5	Kim 2005	6	No significant difference
		Zhao 2017	6	No significant difference
		Mauras 2005	12/24	No significant difference
		Wang 2021	12	Significant increase
		Zeng 2015	12	Significant increase
**HbA1c**	1	Kim 2005	6	No significant difference
**Insulin**	1	Lanes 2006	Unclear	No significant difference
**Insulin resistance**	1	Mauras 2005	12/24	No significant difference
**Insulin sensitivity**	1	Mauras 2005	12/24	No significant difference

HbA1c, hemoglobin A1c

Table S5. Calcium and phosphorus metabolic outcomes between GH treatment and non-medical treatment in GHD children

Specific outcomes	No. of studies	Study ID	Measurement time (month)	Conclusion
**25-OHD**	1	Zeng 2015	12	Significant increase
**BMD**	1	Mauras 2005	12/24	No significant difference

25-OHD, 25-hydroxy vitamin D; BMD, bone mineral density

Table S6. Thyroid hormone outcomes between GH treatment and non-medical treatment in GHD children

Specific outcomes	No. of studies	Study ID	Measurement time (month)	Conclusion
**FT3**	2	Zhang 2021	6	No significant difference
		Wang 2021	12	Significant increase
**FT4**	2	Zhang 2021	6	No significant difference
		Wang 2021	12	No significant difference
**T3**	1	Zhang 2021	6	No significant difference
**T4**	1	Zhang 2021	6	No significant difference
**TSH**	2	Zhang 2021	6	No significant difference
		Wang 2021	12	Significant decrease
**Thyroid level**	1	Zhao 2017	6	No significant difference

FT3, free triiodothyronine; FT4, serum free thyroxine; T3, triiodothyronine; T4, thyroxine; TSH, thyroid-stimulating hormone

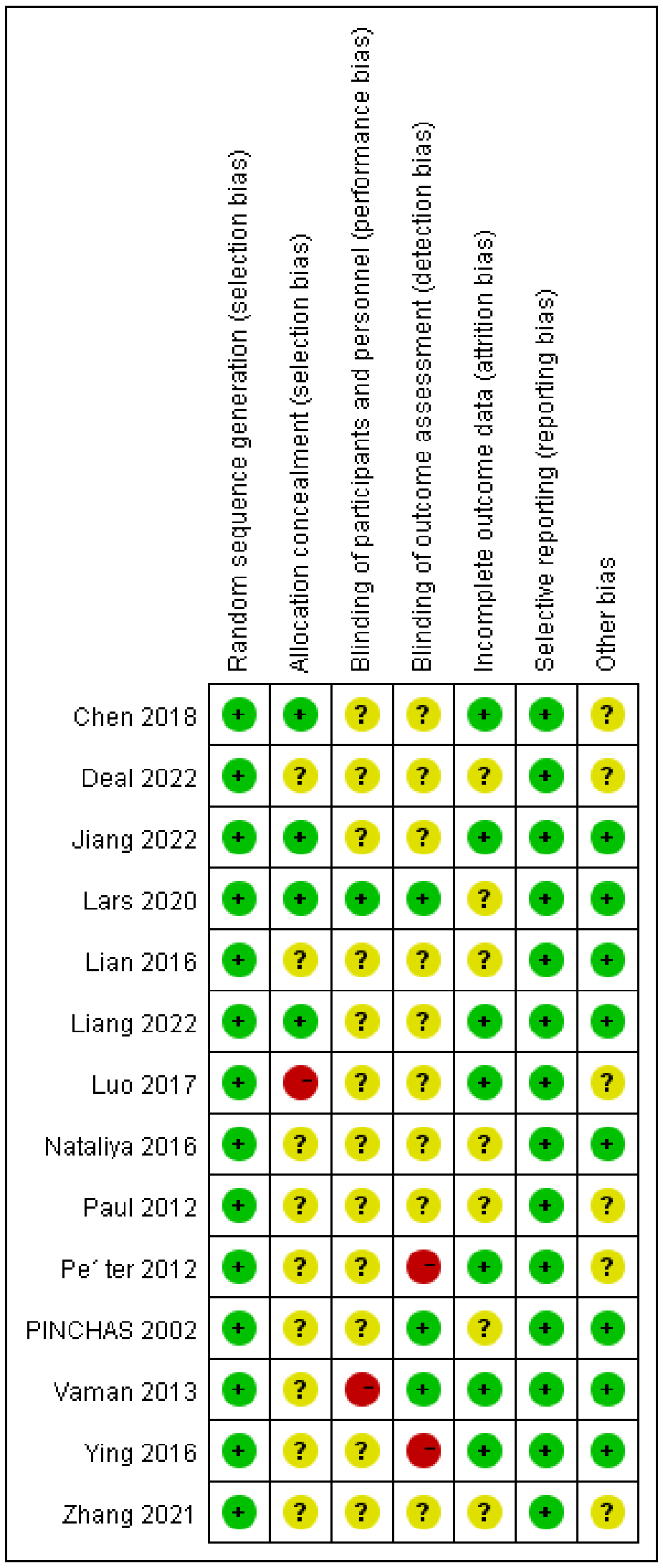

Figure S1. Risk of bias judgements for each study and domain.

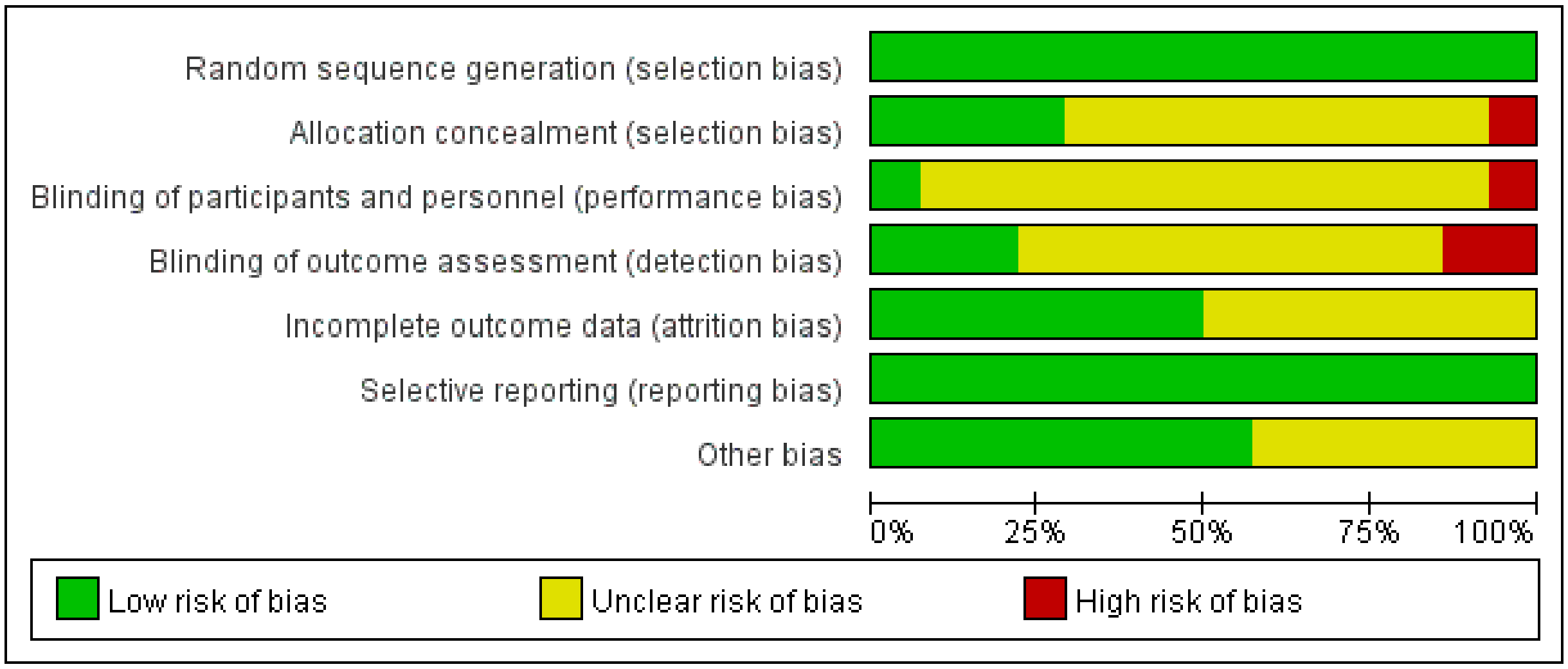

Figure S2. Proportion of studies judged as low, unclear or high risk of bias.

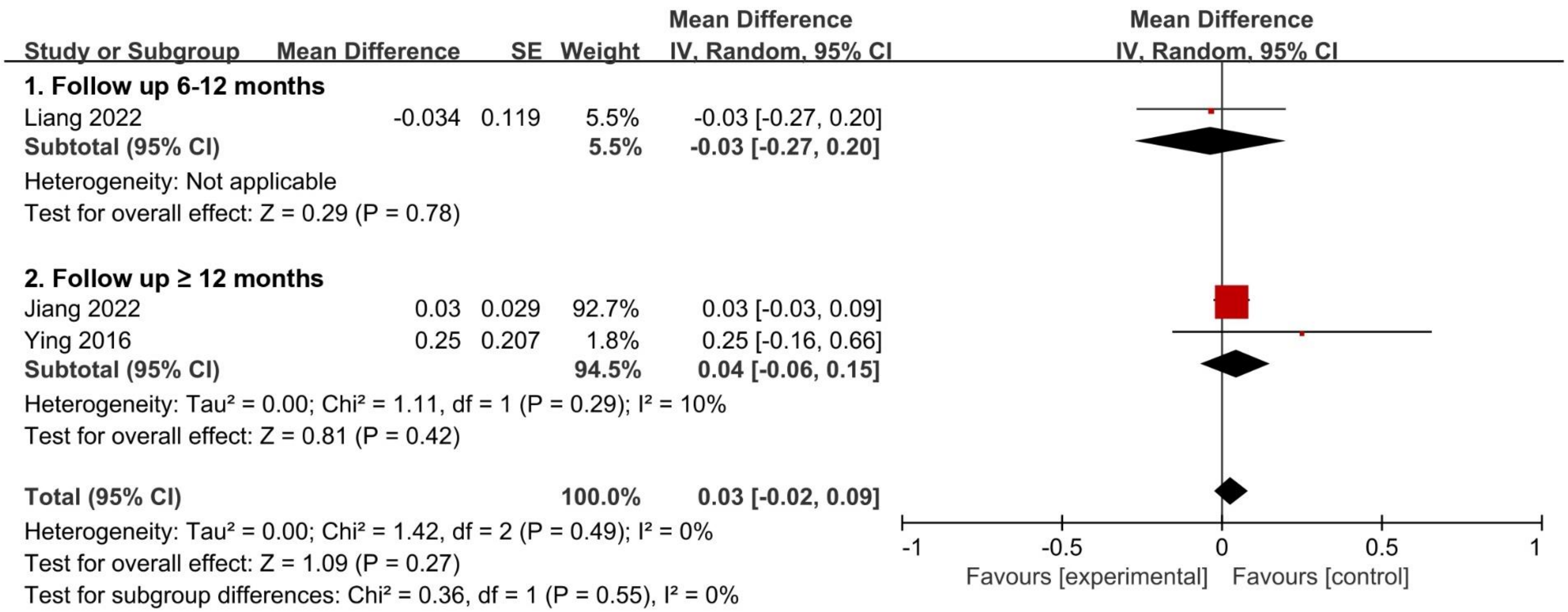

Figure S3. Fasting plasma glucose levels after low-dose GH treatment. CI: confidence interval; IV, inverse variance; SE, standard error.

Study or Subgroup	Mean Difference	SE	Weight	Mean Difference IV, Random, 95% CI
**1. Follow up ≤ 6 months**				
Deal 2022	0.18	0.054	10.1%	0.18 [0.07, 0.29]
Liang 2022	0.352	0.198	5.5%	0.35 [-0.04, 0.74]
**Subtotal (95% CI)**			**15.7%**	**0.19 [0.09, 0.29]**
Heterogeneity: Tau² = 0.00; Chi² = 0.70, df = 1 (P = 0.40); I² = 0%				
Test for overall effect: Z = 3.68 (P = 0.0002)				
**2. Follow up 6-12 months**				
Chen 2018	0.04	0.082	9.3%	0.04 [-0.12, 0.20]
Deal 2022	0.207	0.032	10.6%	0.21 [0.14, 0.27]
Jiang 2022	0.02	0.029	10.6%	0.02 [-0.04, 0.08]
Paul 2012	0.57	0.061	10.0%	0.57 [0.45, 0.69]
Vaman 2013	0.57	0.048	10.3%	0.57 [0.48, 0.66]
Ying 2016	0.53	0.333	2.9%	0.53 [-0.12, 1.18]
**Subtotal (95% CI)**			**53.7%**	**0.30 [0.08, 0.52]**
Heterogeneity: Tau² = 0.06; Chi² = 139.23, df = 5 (P < 0.00001); I² = 96%				
Test for overall effect: Z = 2.71 (P = 0.007)				
**3. Follow up ≥ 12 months**				
Luo 2017	0.41	0.03	10.6%	0.41 [0.35, 0.47]
Paul 2012	0.38	0.062	9.9%	0.38 [0.26, 0.50]
Vaman 2013	0.4	0.059	10.0%	0.40 [0.28, 0.52]
**Subtotal (95% CI)**			**30.6%**	**0.40 [0.36, 0.45]**
Heterogeneity: Tau² = 0.00; Chi² = 0.19, df = 2 (P = 0.91); I² = 0%				
Test for overall effect: Z = 16.44 (P < 0.00001)				
**Total (95% CI)**			**100.0%**	**0.32 [0.19, 0.45]**
Heterogeneity: Tau² = 0.04; Chi² = 182.62, df = 10 (P < 0.00001); I² = 95%				
Test for overall effect: Z = 4.77 (P < 0.00001)				
Test for subgroup differences: Chi² = 13.85, df = 2 (P = 0.0010), I² = 85.6%				

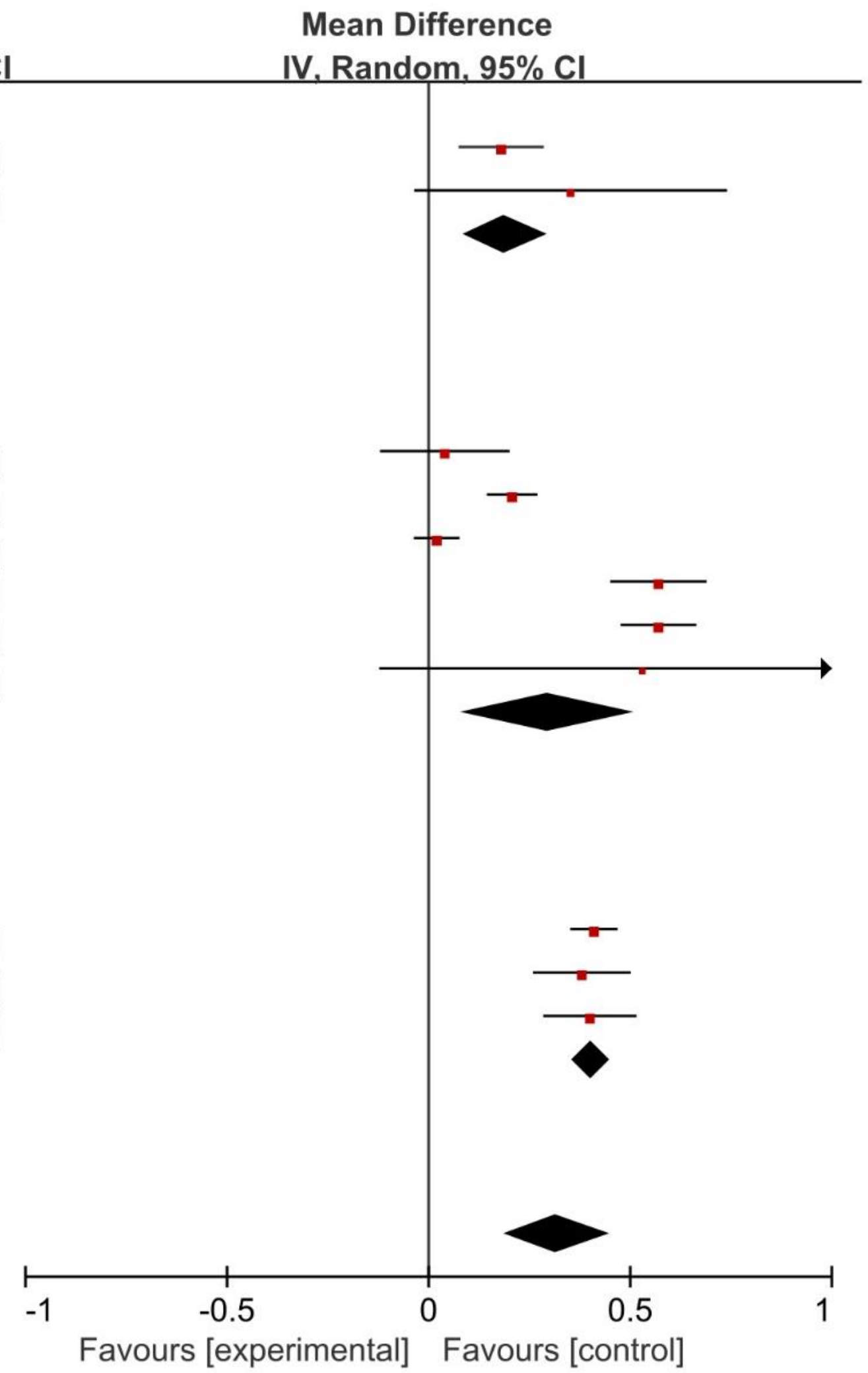

Figure S4. Fasting plasma glucose after medium-dose GH treatment. CI: confidence interval; IV, inverse variance; SE, standard error.

Study or Subgroup	Mean Difference	SE	Weight	Mean Difference IV, Random, 95% CI
**1. Follow up ≤ 6 months**				
Deal 2022	0.15	0.055	11.8%	0.15 [0.04, 0.26]
**Subtotal (95% CI)**			**11.8%**	**0.15 [0.04, 0.26]**
Heterogeneity: Not applicable				
Test for overall effect: Z = 2.73 (P = 0.006)				
**2. Follow up 6-12 months**				
Chen 2018	0.24	0.087	9.3%	0.24 [0.07, 0.41]
Deal 2022	0.254	0.03	13.6%	0.25 [0.20, 0.31]
Paul 2012	0.39	0.073	10.4%	0.39 [0.25, 0.53]
Pe´ ter 2012	0.86	0.166	4.8%	0.86 [0.53, 1.19]
Vaman 2013	0.39	0.058	11.6%	0.39 [0.28, 0.50]
**Subtotal (95% CI)**			**49.6%**	**0.37 [0.25, 0.50]**
Heterogeneity: $Tau^2 = 0.01$; $Chi^2 = 18.12$, df = 4 (P = 0.001); $I^2 = 78\%$				
Test for overall effect: Z = 5.78 (P < 0.00001)				
**3. Follow up ≥ 12 months**				
Luo 2017	0.355	0.044	12.7%	0.35 [0.27, 0.44]
Nataliya 2016	0.32	0.069	10.7%	0.32 [0.18, 0.46]
Paul 2012	0.55	0.073	10.4%	0.55 [0.41, 0.69]
Pe´ ter 2012	0.63	0.167	4.7%	0.63 [0.30, 0.96]
**Subtotal (95% CI)**			**38.5%**	**0.43 [0.30, 0.55]**
Heterogeneity: $Tau^2 = 0.01$; $Chi^2 = 8.50$, df = 3 (P = 0.04); $I^2 = 65\%$				
Test for overall effect: Z = 6.79 (P < 0.00001)				
**Total (95% CI)**			**100.0%**	**0.37 [0.28, 0.45]**
Heterogeneity: $Tau^2 = 0.01$; $Chi^2 = 42.14$, df = 9 (P < 0.00001); $I^2 = 79\%$				
Test for overall effect: Z = 8.30 (P < 0.00001)				
Test for subgroup differences: $Chi^2 = 12.73$, df = 2 (P = 0.002), $I^2 = 84.3\%$				

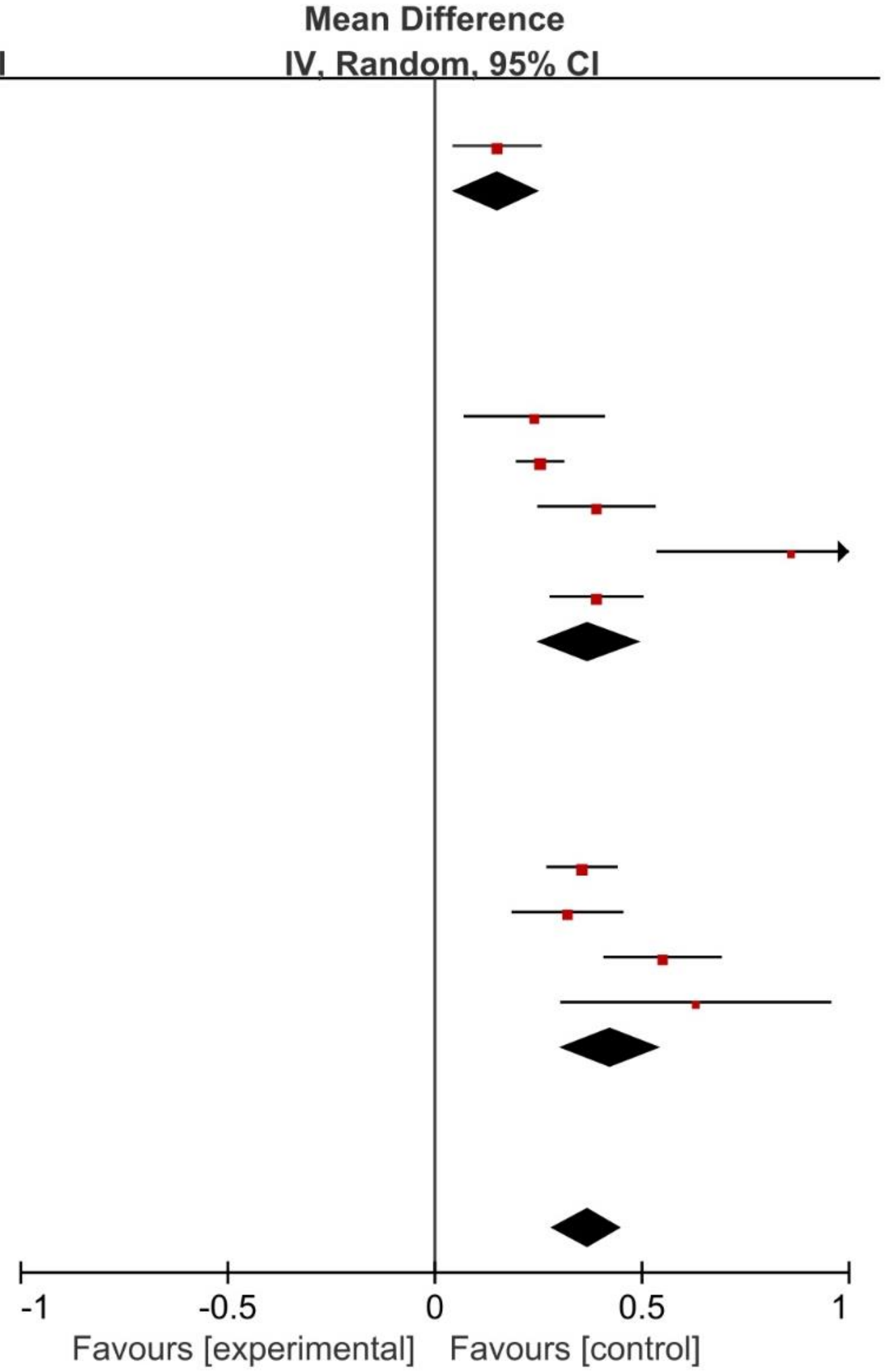

Figure S5. Fasting plasma glucose levels after high-dose GH treatment. CI: confidence interval; IV, inverse variance; SE, standard error.

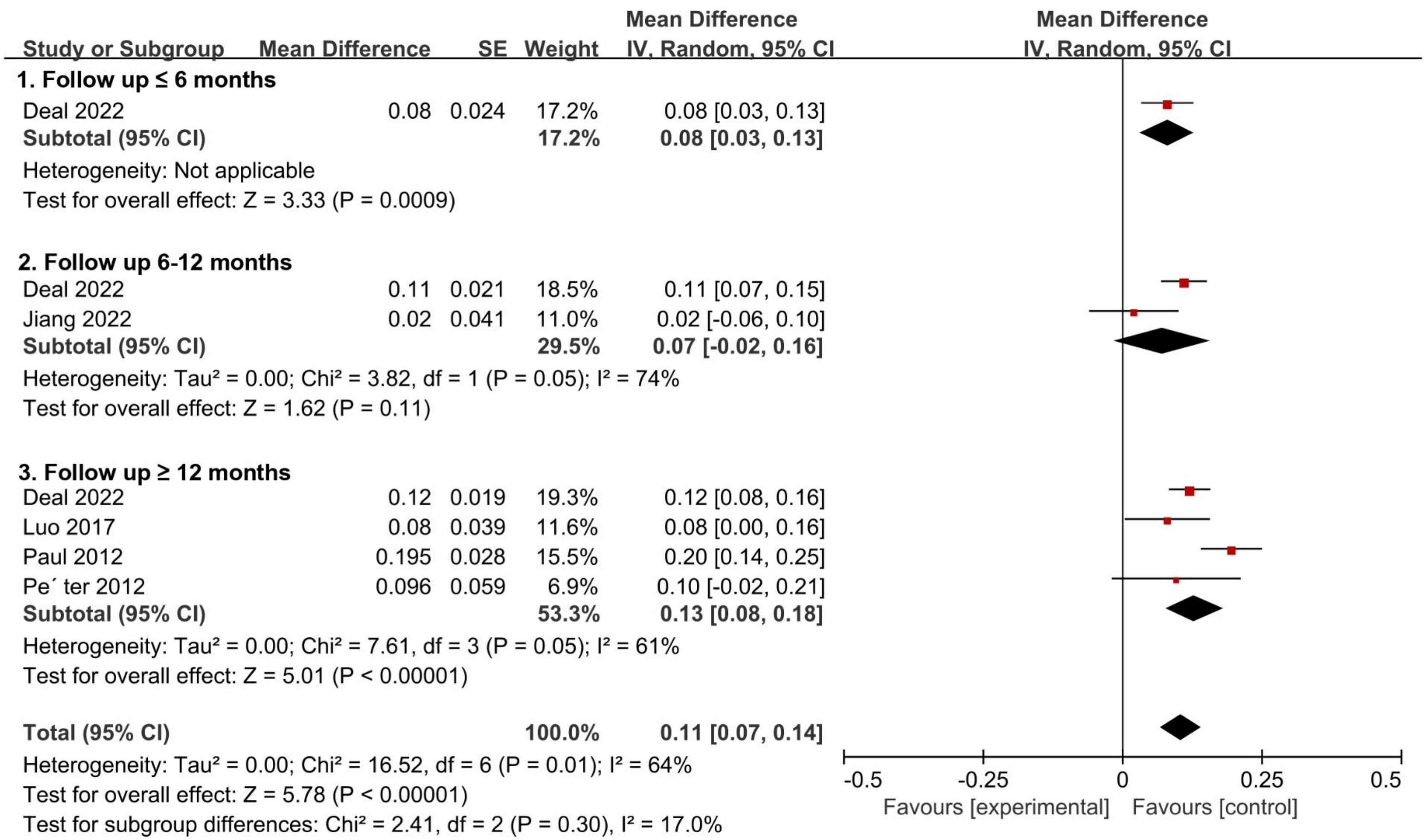

Figure S6. HbA1c levels after medium-dose GH treatment. CI: confidence interval; IV, inverse variance; SE, standard error.

Study or Subgroup	Mean Difference	SE	Weight	Mean Difference IV, Random, 95% CI
**1. Follow up ≤ 6 months**				
Deal 2022	0.091	0.016	20.5%	0.09 [0.06, 0.12]
**Subtotal (95% CI)**			**20.5%**	**0.09 [0.06, 0.12]**
Heterogeneity: Not applicable				
Test for overall effect: Z = 5.69 (P < 0.00001)				
**2. Follow up 6-12 months**				
Deal 2022	0.17	0.022	18.4%	0.17 [0.13, 0.21]
**Subtotal (95% CI)**			**18.4%**	**0.17 [0.13, 0.21]**
Heterogeneity: Not applicable				
Test for overall effect: Z = 7.73 (P < 0.00001)				
**3. Follow up ≥ 12 months**				
Deal 2022	0.18	0.025	17.3%	0.18 [0.13, 0.23]
Luo 2017	0.14	0.063	7.2%	0.14 [0.02, 0.26]
Nataliya 2016	0.064	0.039	12.5%	0.06 [-0.01, 0.14]
Paul 2012	0.17	0.028	16.2%	0.17 [0.12, 0.22]
Pe´ ter 2012	0.067	0.058	8.0%	0.07 [-0.05, 0.18]
**Subtotal (95% CI)**			**61.2%**	**0.13 [0.08, 0.18]**
Heterogeneity: Tau² = 0.00; Chi² = 8.86, df = 4 (P = 0.06); I² = 55%				
Test for overall effect: Z = 5.26 (P < 0.00001)				
**Total (95% CI)**			**100.0%**	**0.13 [0.09, 0.17]**
Heterogeneity: Tau² = 0.00; Chi² = 19.36, df = 6 (P = 0.004); I² = 69%				
Test for overall effect: Z = 6.51 (P < 0.00001)				
Test for subgroup differences: Chi² = 8.75, df = 2 (P = 0.01), I² = 77.1%				

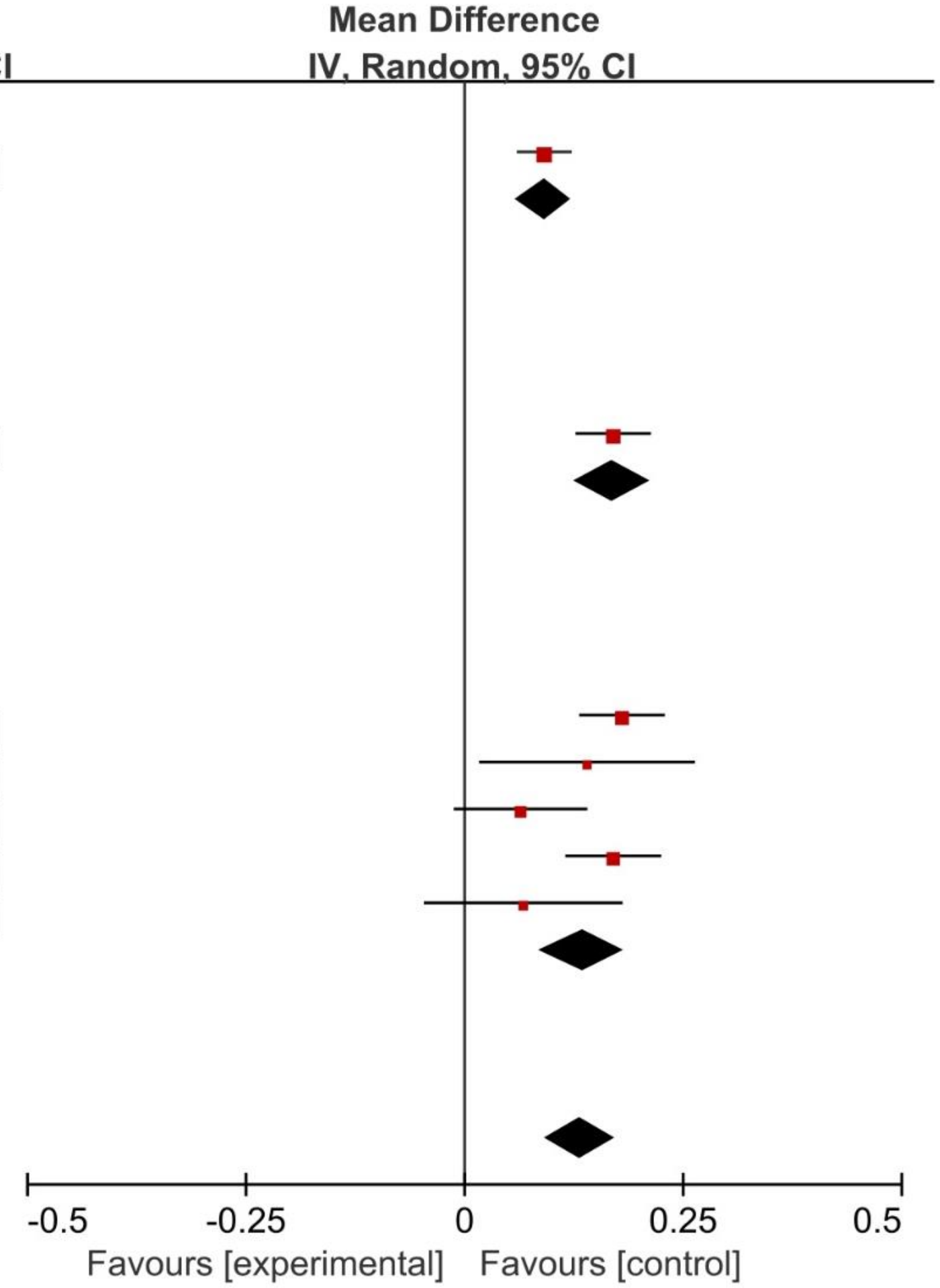

Figure S7. HbA1c levels after high-dose GH treatment. CI: confidence interval; IV, inverse variance; SE, standard error.

Study or Subgroup	Mean Difference	SE	Weight	Mean Difference IV, Random, 95% CI
**1. Follow up ≤ 6 months**				
Deal 2022	1.095	0.253	15.1%	1.09 [0.60, 1.59]
Liang 2022	3.166	2.026	3.7%	3.17 [-0.80, 7.14]
**Subtotal (95% CI)**			**18.8%**	**1.15 [0.47, 1.84]**
Heterogeneity: Tau² = 0.06; Chi² = 1.03, df = 1 (P = 0.31); I² = 3%				
Test for overall effect: Z = 3.33 (P = 0.0009)				
**2. Follow up 6-12 months**				
Deal 2022	1.69	0.36	14.4%	1.69 [0.98, 2.40]
Jiang 2022	0.31	0.259	15.1%	0.31 [-0.20, 0.82]
**Subtotal (95% CI)**			**29.4%**	**0.98 [-0.37, 2.33]**
Heterogeneity: Tau² = 0.85; Chi² = 9.68, df = 1 (P = 0.002); I² = 90%				
Test for overall effect: Z = 1.42 (P = 0.16)				
**3. Follow up ≥ 12 months**				
Deal 2022	1	0.336	14.6%	1.00 [0.34, 1.66]
Lars 2020	3.096	0.93	9.3%	3.10 [1.27, 4.92]
Luo 2017	2.685	0.323	14.6%	2.69 [2.05, 3.32]
PINCHAS 2002	4	0.489	13.3%	4.00 [3.04, 4.96]
**Subtotal (95% CI)**			**51.8%**	**2.64 [1.27, 4.00]**
Heterogeneity: Tau² = 1.66; Chi² = 29.21, df = 3 (P < 0.00001); I² = 90%				
Test for overall effect: Z = 3.79 (P = 0.0002)				
**Total (95% CI)**			**100.0%**	**1.93 [1.06, 2.79]**
Heterogeneity: Tau² = 1.23; Chi² = 69.24, df = 7 (P < 0.00001); I² = 90%				
Test for overall effect: Z = 4.37 (P < 0.0001)				
Test for subgroup differences: Chi² = 4.02, df = 2 (P = 0.13), I² = 50.2%				

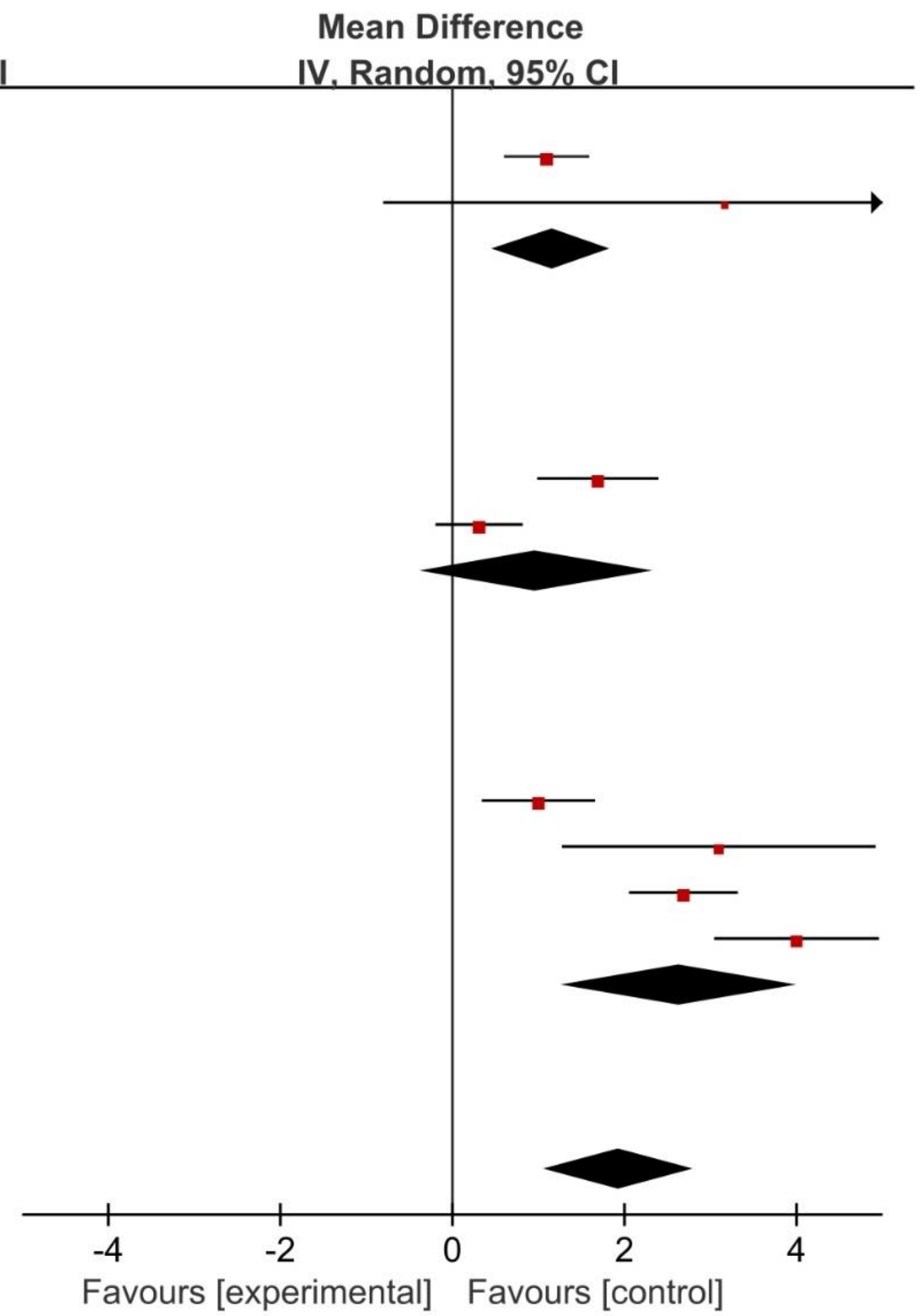

Figure S8. Fasting insulin levels after medium-dose GH treatment. CI: confidence interval; IV, inverse variance; SE, standard error.

Study or Subgroup	Mean Difference	SE	Weight	Mean Difference IV, Random, 95% CI
**1. Follow up ≤ 6 months**				
Deal 2022	1.954	0.202	17.7%	1.95 [1.56, 2.35]
**Subtotal (95% CI)**			**17.7%**	**1.95 [1.56, 2.35]**
Heterogeneity: Not applicable				
Test for overall effect: Z = 9.67 (P < 0.00001)				
**2. Follow up 6-12 months**				
Deal 2022	2.86	0.361	17.4%	2.86 [2.15, 3.57]
**Subtotal (95% CI)**			**17.4%**	**2.86 [2.15, 3.57]**
Heterogeneity: Not applicable				
Test for overall effect: Z = 7.92 (P < 0.00001)				
**3. Follow up ≥ 12 months**				
Deal 2022	2.98	0.36	17.4%	2.98 [2.27, 3.69]
Luo 2017	3.52	0.534	16.9%	3.52 [2.47, 4.57]
Nataliya 2016	1.067	1.297	13.4%	1.07 [-1.48, 3.61]
PINCHAS 2002	8	0.409	17.3%	8.00 [7.20, 8.80]
**Subtotal (95% CI)**			**64.9%**	**4.00 [1.09, 6.92]**
Heterogeneity: Tau² = 8.32; Chi² = 100.65, df = 3 (P < 0.00001); I² = 97%				
Test for overall effect: Z = 2.69 (P = 0.007)				
**Total (95% CI)**			**100.0%**	**3.48 [1.62, 5.34]**
Heterogeneity: Tau² = 5.03; Chi² = 179.31, df = 5 (P < 0.00001); I² = 97%				
Test for overall effect: Z = 3.67 (P = 0.0002)				
Test for subgroup differences: Chi² = 6.30, df = 2 (P = 0.04), I² = 68.2%				

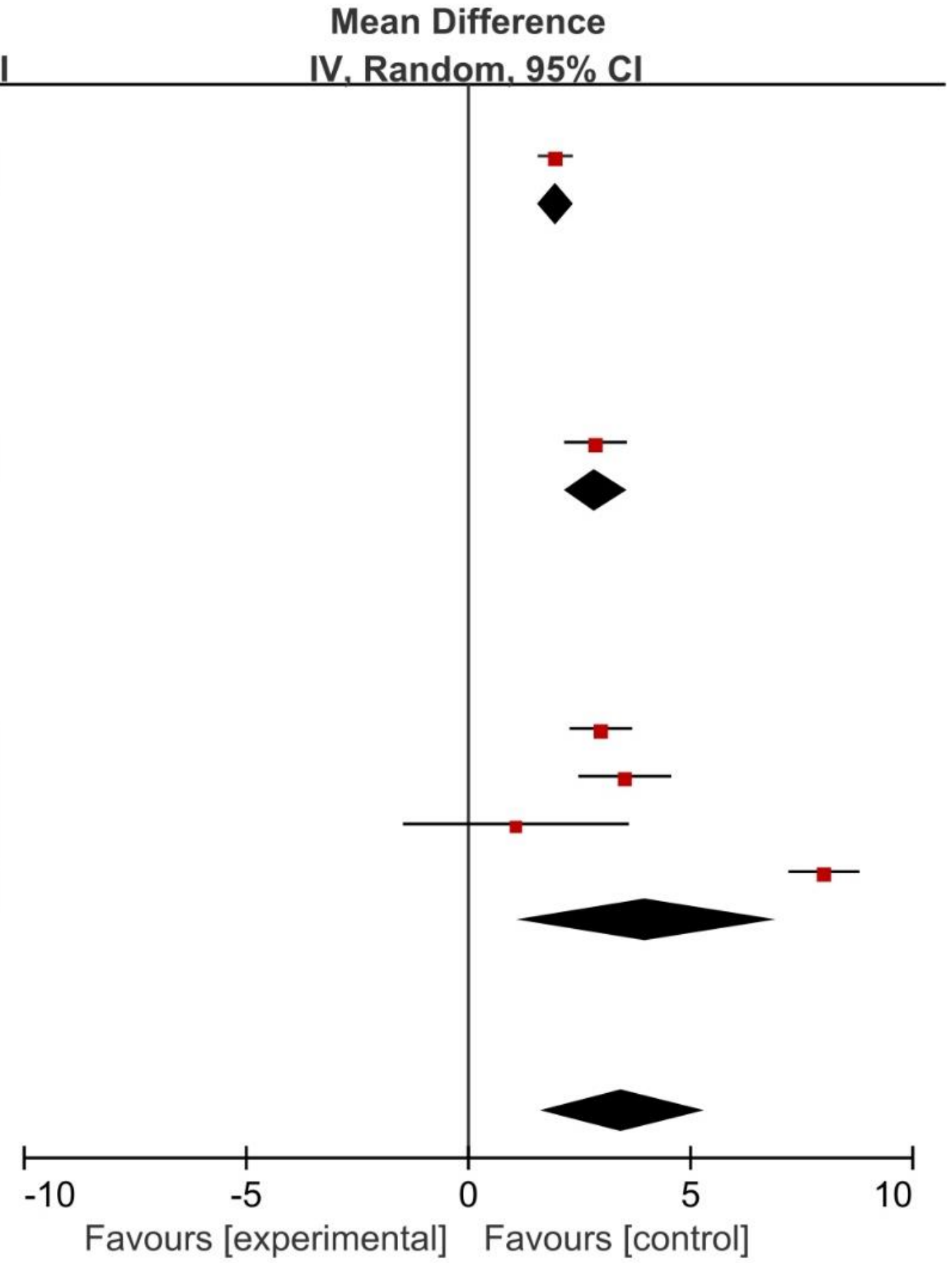

Figure S9. Fasting insulin levels after high-dose GH treatment. CI: confidence interval; IV, inverse variance; SE, standard error.

Study or Subgroup	Mean Difference	SE	Weight	Mean Difference IV, Random, 95% CI
**1. Follow up ≤ 6 months**				
Liang 2022	-0.253	0.253	11.0%	-0.25 [-0.75, 0.24]
**Subtotal (95% CI)**			**11.0%**	**-0.25 [-0.75, 0.24]**
Heterogeneity: Not applicable				
Test for overall effect: Z = 1.00 (P = 0.32)				
**2. Follow up 6-12 months**				
Jiang 2022	0.06	0.046	22.7%	0.06 [-0.03, 0.15]
**Subtotal (95% CI)**			**22.7%**	**0.06 [-0.03, 0.15]**
Heterogeneity: Not applicable				
Test for overall effect: Z = 1.30 (P = 0.19)				
**3. Follow up ≥ 12 months**				
Chen 2018	-0.53	0.06	22.1%	-0.53 [-0.65, -0.41]
Luo 2017	-0.235	0.045	22.7%	-0.23 [-0.32, -0.15]
Paul 2012	-0.185	0.072	21.5%	-0.18 [-0.33, -0.04]
**Subtotal (95% CI)**			**66.4%**	**-0.32 [-0.52, -0.11]**
Heterogeneity: $Tau^2 = 0.03$; $Chi^2 = 19.24$, df = 2 (P < 0.0001); $I^2 = 90\%$				
Test for overall effect: Z = 3.04 (P = 0.002)				
**Total (95% CI)**			**100.0%**	**-0.22 [-0.45, 0.00]**
Heterogeneity: $Tau^2 = 0.06$; $Chi^2 = 62.71$, df = 4 (P < 0.00001); $I^2 = 94\%$				
Test for overall effect: Z = 1.96 (P = 0.05)				
Test for subgroup differences: $Chi^2 = 11.92$, df = 2 (P = 0.003), $I^2 = 83.2\%$				

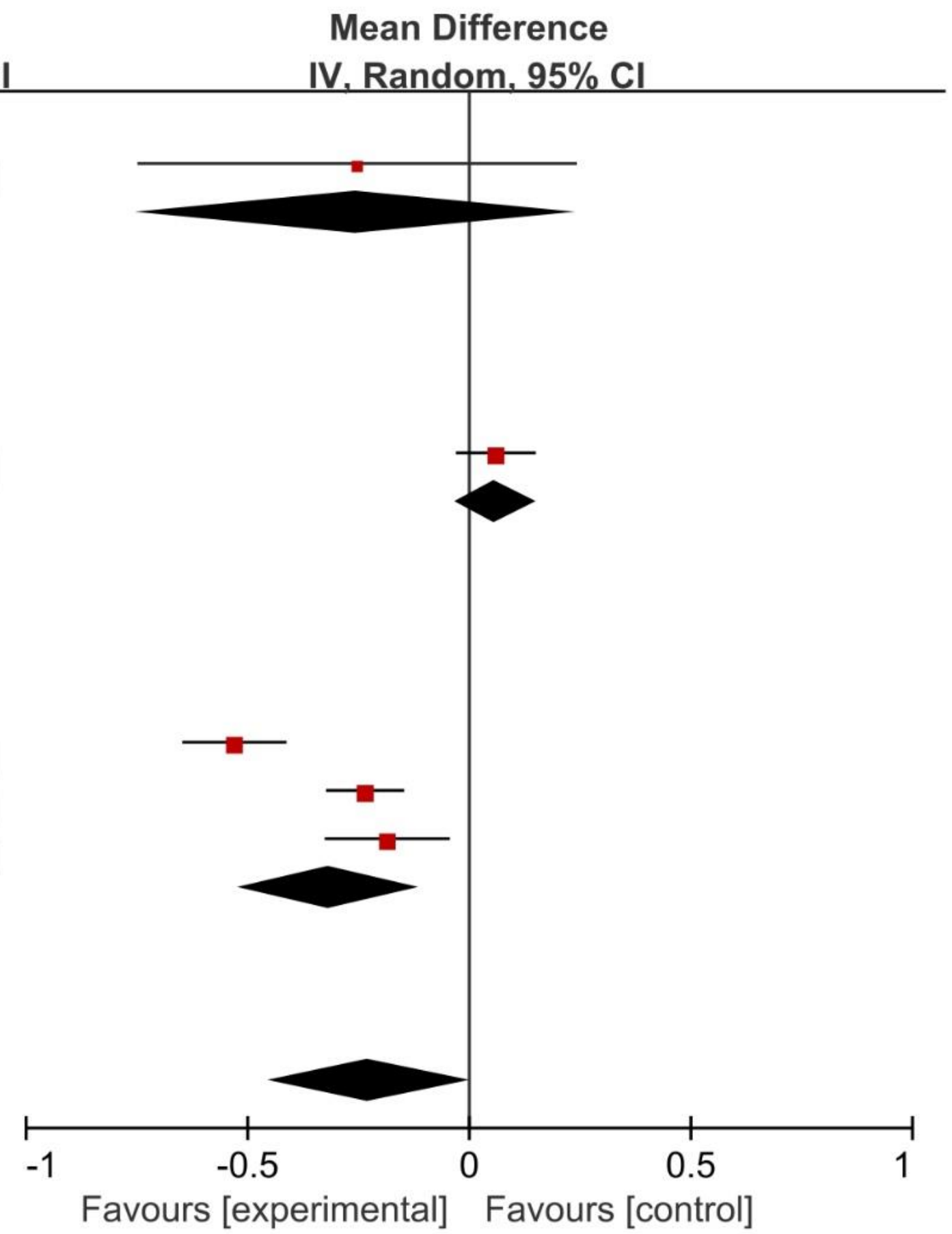

Figure S10. TC levels after medium-dose GH treatment. CI: confidence interval; IV, inverse variance; SE, standard error.

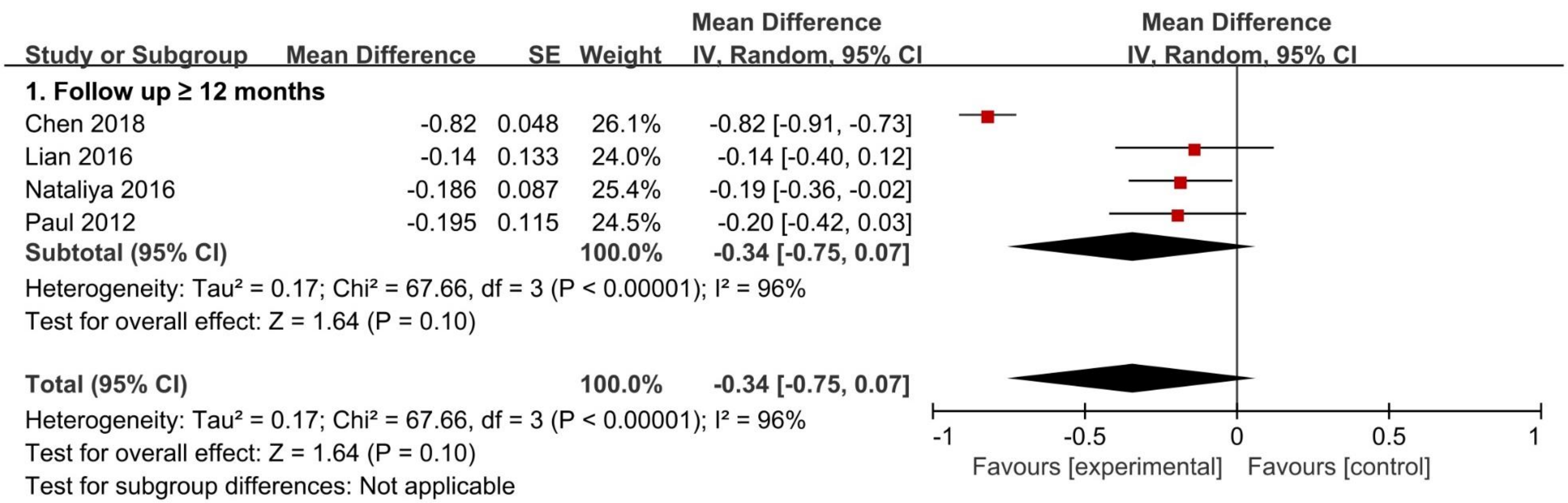

Figure S11. TC levels after high-dose GH treatment. CI: confidence interval; IV, inverse variance; SE, standard error.

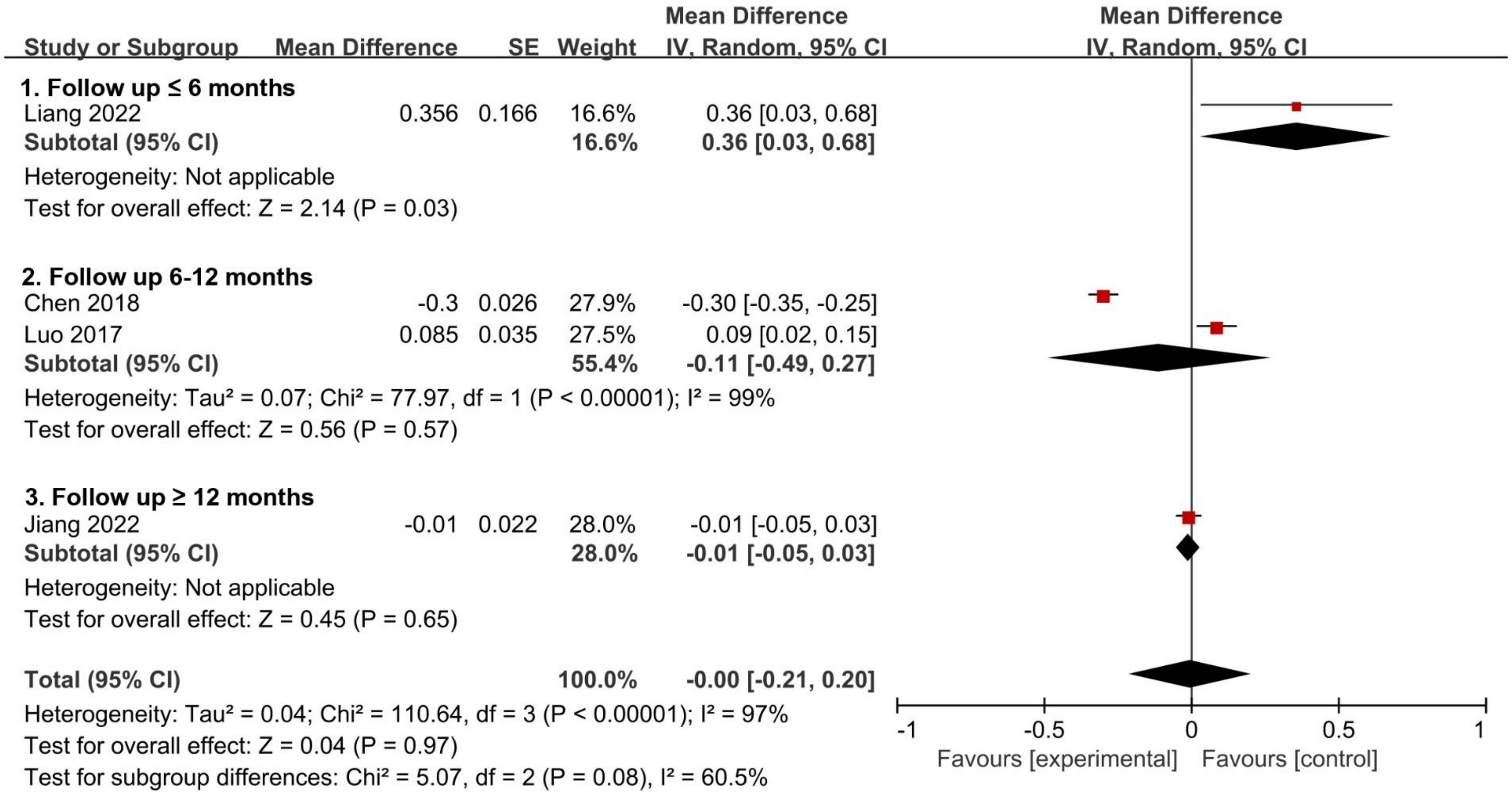

Figure S12. TG levels after medium-dose GH treatment. CI: confidence interval; IV, inverse variance; SE, standard error.

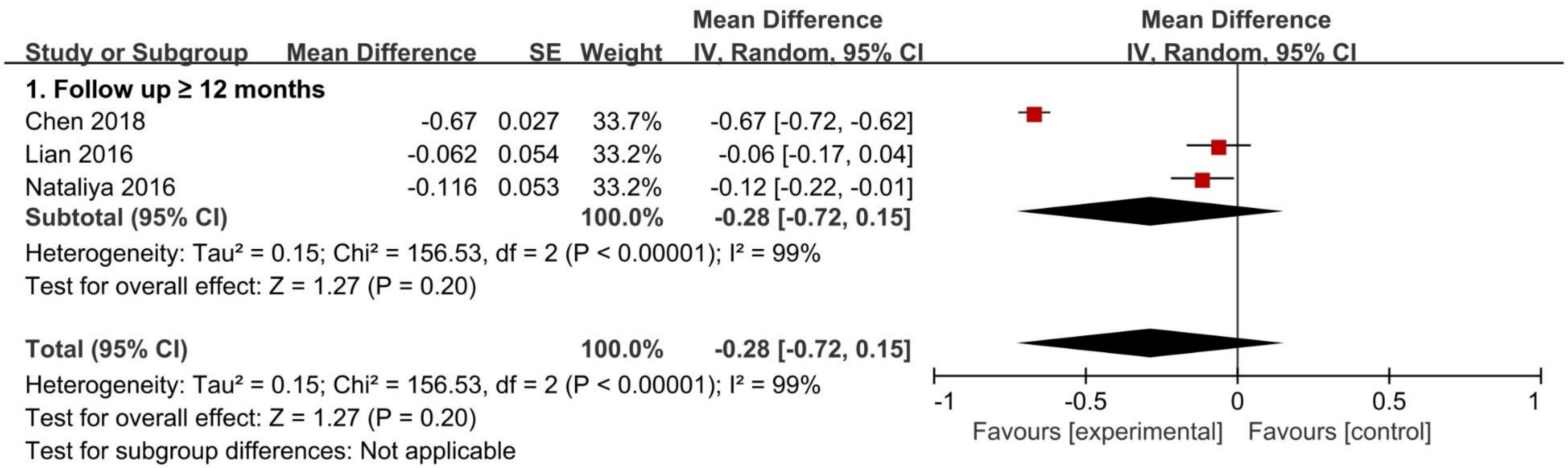

Figure S13. TG levels after high-dose GH treatment. CI: confidence interval; IV, inverse variance; SE, standard error.

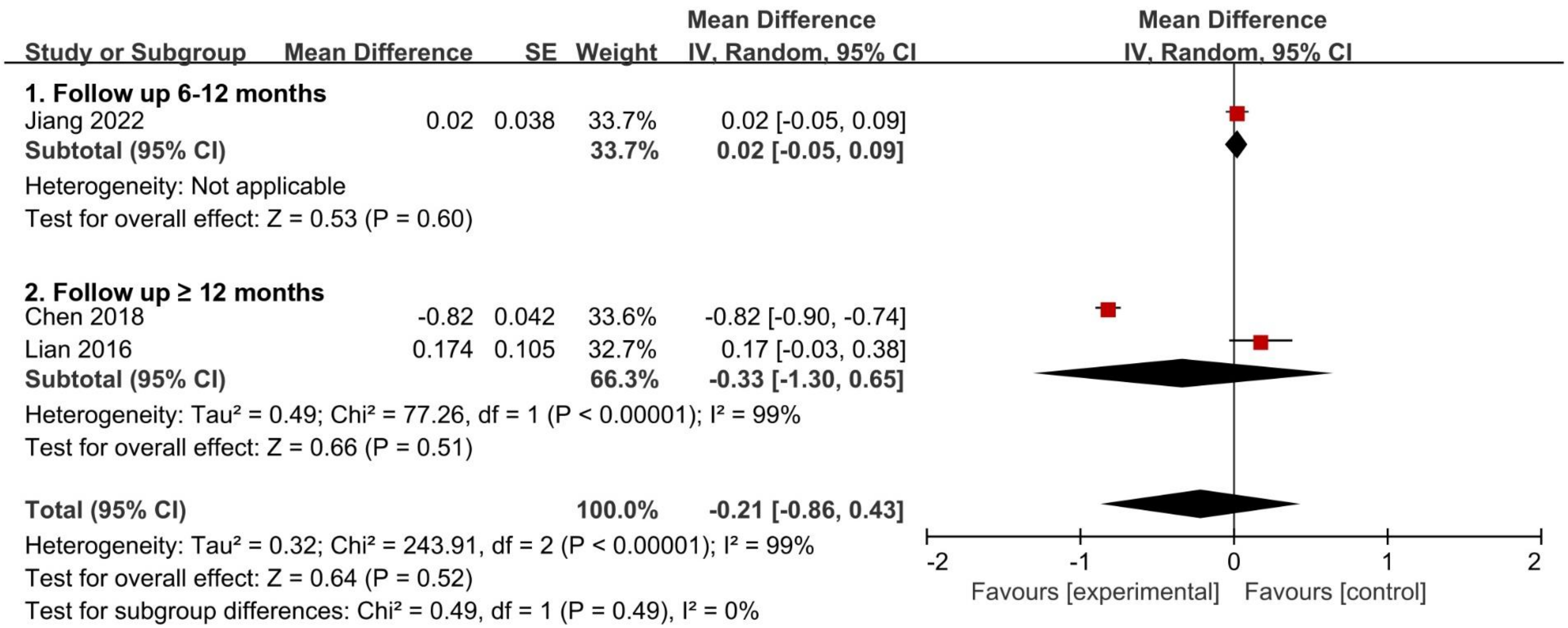

Figure S14. LDL-C after medium-dose GH treatment. CI: confidence interval; IV, inverse variance; SE, standard error.

Study or Subgroup	Mean Difference	SE	Weight	Mean Difference IV, Random, 95% CI
**1. Follow up ≥ 12 months**				
Chen 2018	-0.98	0.048	50.1%	-0.98 [-1.07, -0.89]
Nataliya 2016	-0.293	0.06	49.9%	-0.29 [-0.41, -0.18]
**Subtotal (95% CI)**			**100.0%**	**-0.64 [-1.31, 0.04]**

Heterogeneity: $Tau^2 = 0.23$; $Chi^2 = 79.94$, df = 1 ($P < 0.00001$); $I^2 = 99\%$
Test for overall effect: Z = 1.86 (P = 0.06)

**Total (95% CI)**			**100.0%**	**-0.64 [-1.31, 0.04]**

Heterogeneity: $Tau^2 = 0.23$; $Chi^2 = 79.94$, df = 1 ($P < 0.00001$); $I^2 = 99\%$
Test for overall effect: Z = 1.86 (P = 0.06)
Test for subgroup differences: Not applicable

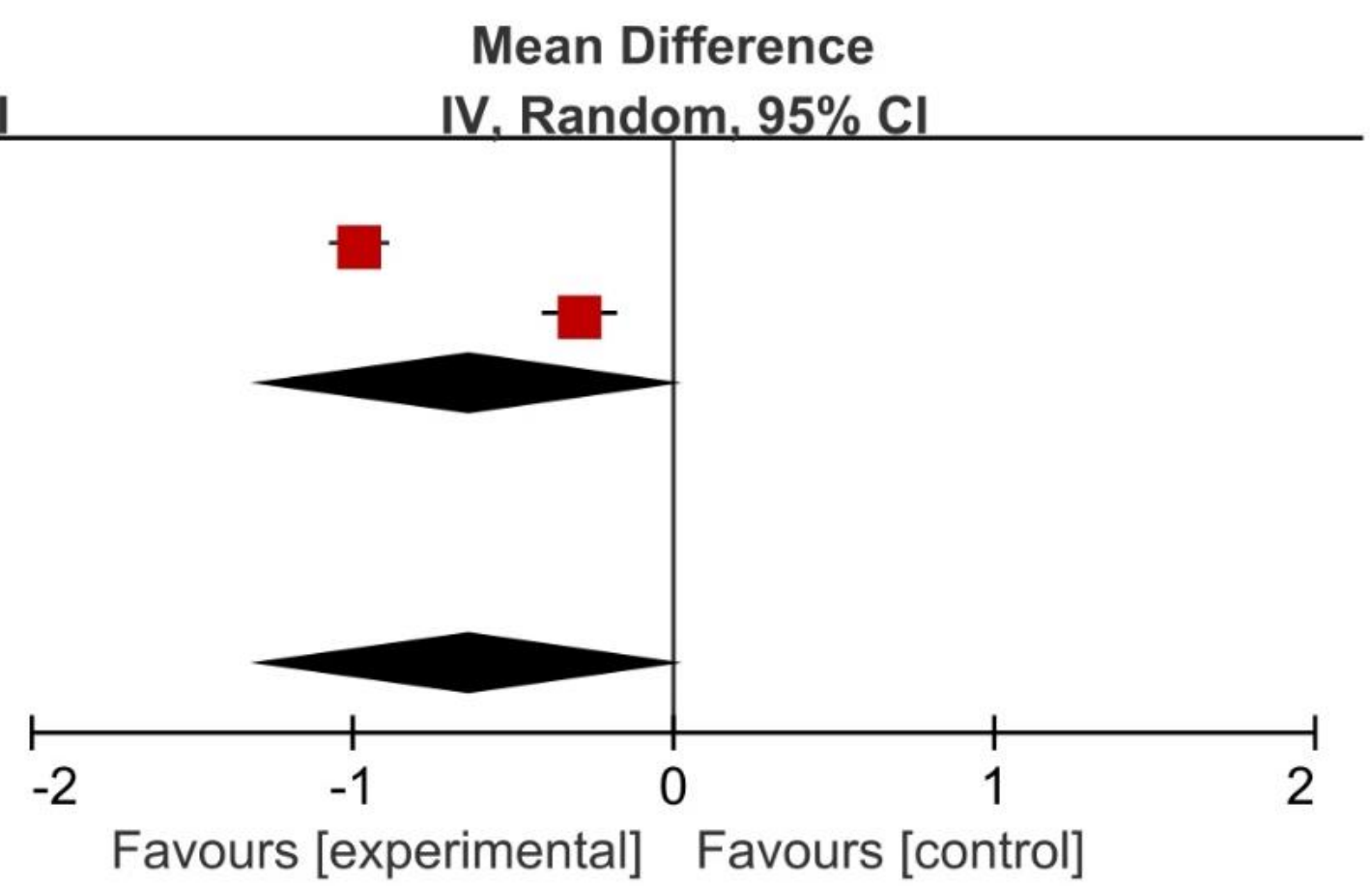

Figure S15. LDL-C levels after high-dose GH treatment. CI: confidence interval; IV, inverse variance; SE, standard error.

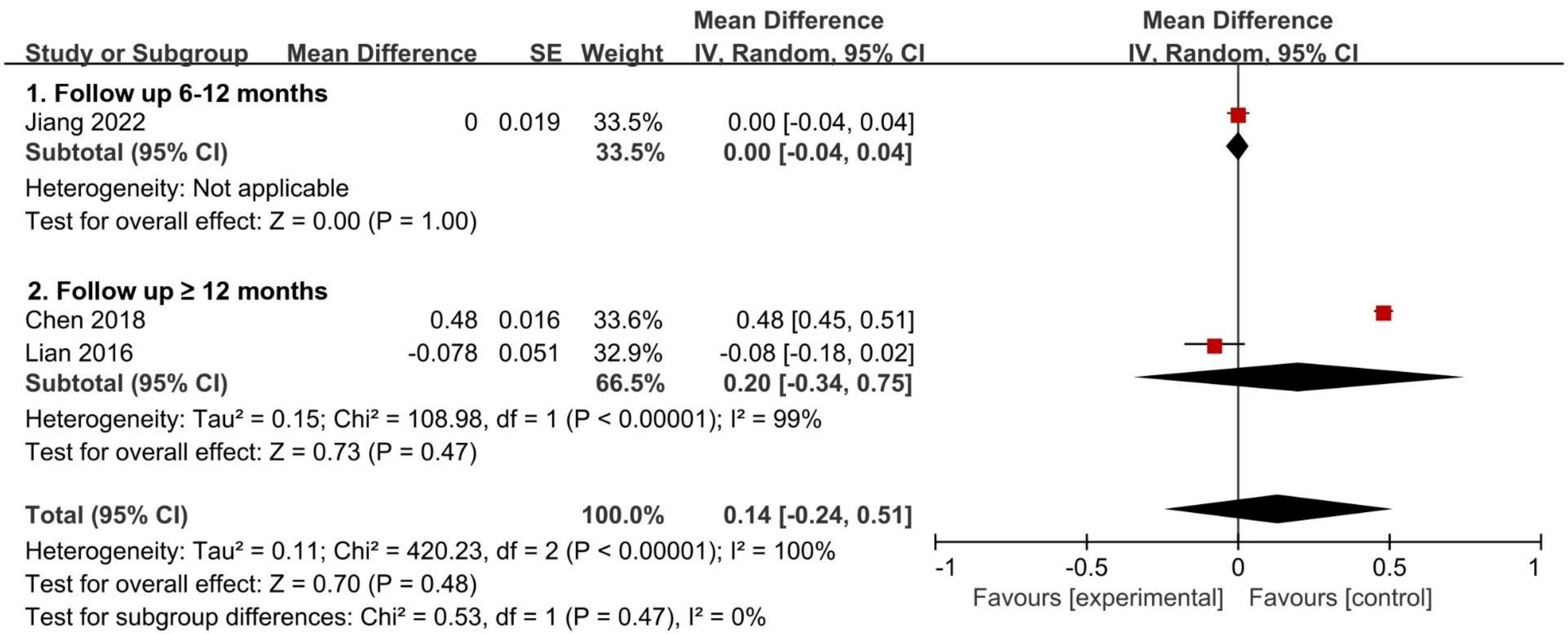

Figure S16. HDL-C after medium-dose GH treatment. CI: confidence interval; IV, inverse variance; SE, standard error.

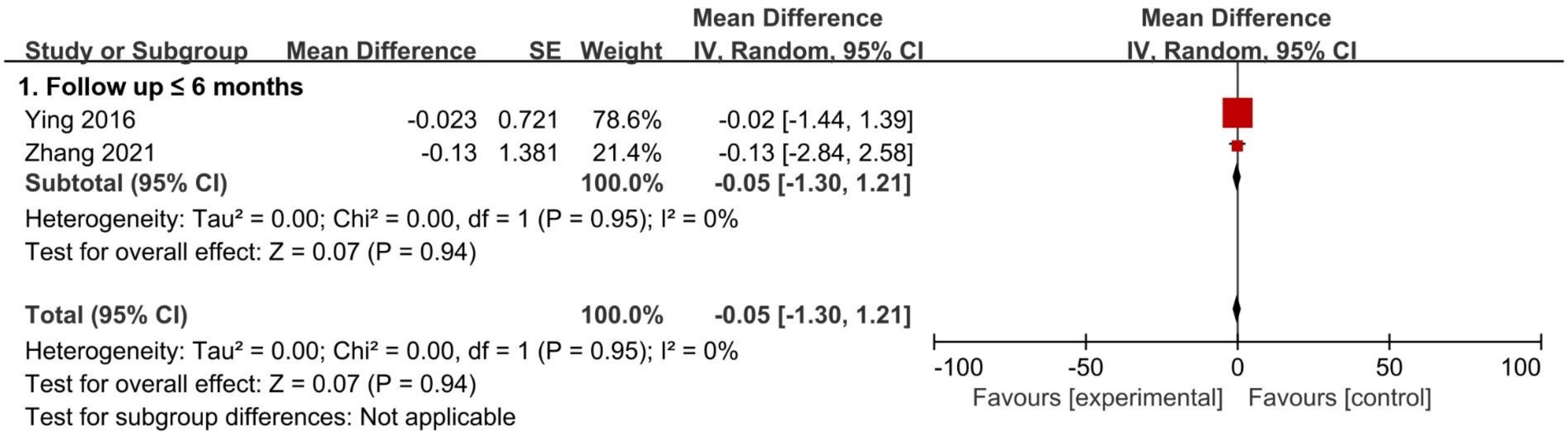

Figure S17. Thyroid-stimulating hormone levels after low-dose GH treatment. CI: confidence interval; IV, inverse variance; SE, standard error.
